# PI3Kα-regulated gelsolin activity is a critical determinant of cardiac cytoskeletal remodeling and heart disease

**DOI:** 10.1038/s41467-018-07812-8

**Published:** 2018-12-19

**Authors:** Vaibhav B. Patel, Pavel Zhabyeyev, Xueyi Chen, Faqi Wang, Manish Paul, Dong Fan, Brent A. McLean, Ratnadeep Basu, Pu Zhang, Saumya Shah, John F. Dawson, W. Glen Pyle, Mousumi Hazra, Zamaneh Kassiri, Saugata Hazra, Bart Vanhaesebroeck, Christopher A. McCulloch, Gavin Y. Oudit

**Affiliations:** 1 Division of Cardiology, Department of Medicine, 2C2, 8440-112 St, Edmonton, AB T6G 2B7 Canada; 2 Mazankowski Alberta Heart Institute, University of Alberta, 2C2, 8440-112 St, Edmonton, AB T6G 2B7 Canada; 3 Department of Biotechnology, North Orissa University, Baripada, 757003 Odisha India; 4 Department of Physiology, University of Alberta, HMRC-407, 116 St 85 Ave, Edmonton, AB T6G 2S2 Canada; 5 Department of Molecular and Cellular Biology, University of Guelph, Guelph, ON N1G 2W1 Canada; 6 Centre of Cardiovascular Investigations, University of Guelph, Guelph, ON N1G 2W1 Canada; 7 Department of Biomedical Sciences, University of Guelph, Guelph, ON N1G 2W1 Canada; 8 Department of Botany and Microbiology, Gurukula Kangri University, Haridwar, 249404 Uttarakhand India; 9 Department of Biotechnology, Indian Institute of Technology, Roorkee, 247667 Uttarakhand India; 10 Centre for Nanotechnology, Indian Institute of Technology Roorkee, Roorkee, 247667 Uttarakhand India; 11 UCL Cancer Institute, University College London, London, WC1E 6BT England, UK; 12 Matrix Dynamics Group, Faculty of Dentistry, University of Toronto, Toronto, ON M5S 3E2 Canada; 13 Present Address: Department of Physiology and Pharmacology and Libin Cardiovascular Institute of Alberta, Cumming School of Medicine, University of Calgary, HMRB-71, 3330 Hospital Drive NW, Calgary, AB T2N 4N1 Canada

## Abstract

Biomechanical stress and cytoskeletal remodeling are key determinants of cellular homeostasis and tissue responses to mechanical stimuli and injury. Here we document the increased activity of gelsolin, an actin filament severing and capping protein, in failing human hearts. Deletion of gelsolin prevents biomechanical stress-induced adverse cytoskeletal remodeling and heart failure in mice. We show that phosphatidylinositol (3,4,5)-triphosphate (PIP3) lipid suppresses gelsolin actin-severing and capping activities. Accordingly, loss of PI3Kα, the key PIP3-producing enzyme in the heart, increases gelsolin-mediated actin-severing activities in the myocardium in vivo, resulting in dilated cardiomyopathy in response to pressure-overload. Mechanical stretching of adult PI3Kα-deficient cardiomyocytes disrupts the actin cytoskeleton, which is prevented by reconstituting cells with PIP3. The actin severing and capping activities of recombinant gelsolin are effectively suppressed by PIP3. Our data identify the role of gelsolin-driven cytoskeletal remodeling in heart failure in which PI3Kα/PIP3 act as negative regulators of gelsolin activity.

## Abstract (web summary)

Gelsolin is an actin severing and capping protein that regulates cytoskeletal remodeling. Here the authors show that gelsolin is negatively regulated in the heart by PI3Kα‐ generated PIP3, and that loss of gelsolin activity prevents adverse cytoskeletal remodeling and heart failure.

## Introduction

Heart failure (HF) is driven by a complex series of signaling and injury pathways that lead to maladaptive cardiac remodeling^1,2^. Hypertension, which leads to increased afterload and biomechanical stress on the heart, is the most important cause of HF^2,3^. Biomechanical stress is converted to intracellular signals through mechanotransduction processes^4–6^; remodeling of the cytoskeleton is a central feature of these processes. However, the regulation of these processes and their contribution to HF is poorly understood. Gelsolin is a Ca^2+^-regulated actin filament severing and capping protein, that is widely expressed in a variety of tissues including the heart, brain, immune cells, and various cancer tissues^7^. Importantly, gelsolin favors actin depolymerization by virtue of both its actin-severing activity and its ability to cap the barbed ends of actin filaments, resulting in reduced actin polymerization. Gelsolin has a high-positive charge and contains multiple binding sites for Ca^2+^ and phosphatidylinositol lipids^7,8^.

Phosphoinositide 3-kinase (PI3K) activity plays a key role in cell signaling, cell survival, and growth and modulates myocardial contractility^9–11^. Among the eight isoforms of PI3K, the class I PI3Ks isoforms, p110α, β, γ, and δ, which occur in a complex with a regulatory subunit (the complexes are further referred to as PI3Kα, PI3Kβ, PI3Kγ, and PI3Kδ), convert phosphatidylinositol(4,5)-bisphosphate (PIP2) lipid to phosphatidylinositol (3,4,5)-trisphosphate (PIP3). Whereas p110α and p110β show a broad tissue distribution, the expression of p110γ and p110δ is highly enriched in leukocytes, with low levels expressed in other tissues^12^. PIP3 is degraded to PIP2 by the phosphatase and tensin homolog (PTEN) lipid phosphatase^10^. In the heart, both PI3Kα and PI3Kγ control distinct aspects of cardiac structure and function^9,10,13–15^. Exercise and agonizts known to activate PI3Kα are linked to protection from HF^16,17^ while the loss of cardiomyocyte PTEN and enhanced PI3Kα action^10^ protect the heart from damage caused by biomechanical stress^18^.

Using a combination of explanted human and canine hearts, genetic mouse models, computer modeling, and biochemical studies, we identify gelsolin-mediated actin cytoskeletal remodeling as a critical response to biomechanical stress-induced mechanotransduction and in the pathogenesis of HF. We show that gelsolin’s severing activity is inhibited by the PI3Kα product, PIP3, in response to stress-induced cardiac mechanotransduction thereby identifying a central regulatory mechanism of gelsolin’s action. We also highlight the importance of biomechanical stress-induced cytoskeletal remodeling as an essential response involved in adaptive cardiac remodeling.

## Results

### Loss of gelsolin reverses cytoskeletal remodeling and HF

**Fig. 1:**
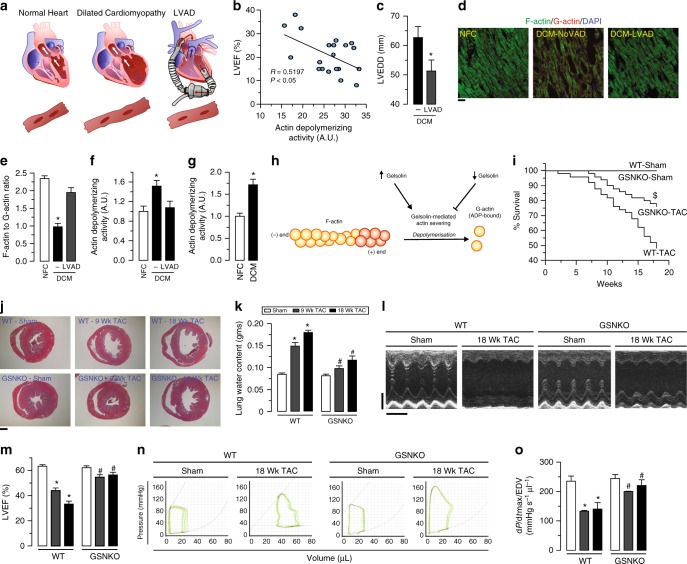
Relationship between gelsolin and adverse cytoskeletal remodeling in DCM and in biomechanical stress-induced HF. **a** Schematic showing pathogenic and reverse remodeling process. Pathogenic remodeling in response to chronic injury leading to ventricular dilation, resulting in HF with reduced EF. LVAD placement results in reverse myocardial remodeling and improved cardiac function. **b** LVEF inversely correlates with myocardial gelsolin actin-depolymerizing activity in humans with DCM; *n* = 20 DCM hearts; age 52.3 ± 2.49 y; 16 male/4 female. **c** LV end-diastolic dimensions (LVEDD) from patients with DCM showing cardiac reverse remodeling in response to LVAD placement. **d**–**g** Representative images of F- and G-actin staining (**d**), F-actin to G-actin ratio (**e**), and actin-depolymerizing activity in human (**f**) and canine (**g**) hearts showing increased actin depolymerization in DCM samples compared with NFC. LVAD placement reduced actin-depolymerizing activity and recovered F to G-actin ratio. **h** Schematic showing the role of gelsolin in actin depolymerization. **i** Kaplan–Meier survival curve showing markedly increased mortality in response to pressure overload for 18 weeks in WT mice. Loss of gelsolin significantly decreased mortality in response to pressure overload. **j** Masson trichrome staining showing increased ventricular dilation in WT mice, which was attenuated in GSNKO mice. **k** Lung water content showing increased pulmonary edema in pressure-overloaded WT mice, which was attenuated in the GSNKO mice. **l**–**o** M-mode echocardiography images (**l**), quantification of LVEF (**m**), representative PV loop images (**n**), and d*p*/d*t*_max_/EDV (load-independent index of systolic function; **o**) showing severe HF with EF in WT mice in response to pressure overload-induced biomechanical stress. Loss of gelsolin markedly preserved cardiac function. Data represent means ± s.e.m. ^*^P < 0.05 compared with the respective control groups (NFC or Sham), ^#^*P* < 0.05 compared with respective WT—9 Wk or 18 week TAC group as determined by unpaired two-tailed Student’s *t* test (**c**, **g**) and one-way ANOVA analysis (**e**, **f**, **k**, **m**, **o**). ^$^*P* < 0.05 for Kaplan–Meir survival analysis (**i**) as determined by log-rank test. Biological replicates: *n* = 20 (**b**), *n* = 8 (**c**–**e**, **n**–**o**), *n* = 6 (**g**), *n* = 50 (**i**), *n* = 4 (**j**) and *n* = 12 (**k**–**m**). Scale bars show 25 µm (**d**), 1 mm (**j**), 2 mm (*y*-axis of **l**), and 200 ms (*x*-axis of **l**)

To screen for novel pathogenic pathways of HF, we utilized explanted failing human hearts with dilated cardiomyopathy (DCM) and assessed the impact of mechanical unloading by the use of left-ventricular (LV) assist devices (LVAD) (Fig. 1a). We found that disease progression in human DCM, as measured by LV ejection fraction (LVEF), is linked to greater gelsolin actin-depolymerizing activity (Fig. 1b and Supplementary Fig. 1a). Interestingly, LVAD therapy improved adverse cytoskeletal remodeling as illustrated by normalization of a decreased F/G-actin ratio and restored the increased actin-depolymerizing activity to basal values (Fig. 1c–f, Supplementary Fig. 1b, Supplementary Data 1). These results illustrate the key sensitivity of gelsolin to mechanical unloading and its relevance in human HF. Canine hearts with naturally occurring DCM showed a similar increase in actin-depolymerizing activity as seen in human DCM (Fig. 1f–g and Supplementary Data 1) implying a conserved mechanism for DCM. Given that gelsolin is a major mediator of actin cytoskeleton remodeling, we hypothesized that this protein could be a critical mediator of HF (Fig. 1h and Supplementary Fig. 2a). In response to advanced pressure-overload, gelsolin-knockout (GSNKO) mice, which do not show any detectable difference in the normal state as compared to wild-type (WT) mice, had markedly reduced HF-related mortality compared with WT mice (Fig. 1i), correlating with reduced ventricular dilation (Fig. 1j) and pulmonary edema (Fig. 1k and Supplementary Data 1). Echocardiographic analysis revealed marked diastolic and systolic dysfunction characteristic of advanced HF in pressure-overloaded WT mice, which were markedly attenuated in GSNKO mice (Fig. 1l, m, Supplementary Fig. 3 and Supplementary Data 1). We also performed load-independent invasive pressure–volume loop analysis which demonstrated a marked protective effect of gelsolin deficiency against the progression to advanced HF in GSNKO mice compared with WT mice (Fig. 1n–o, Supplementary Table 1 and Supplementary Data 1).

**Fig. 2:**
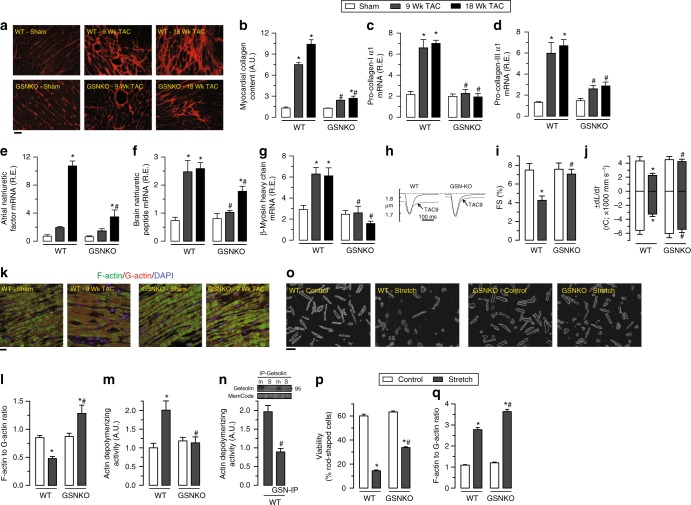
Loss of gelsolin attenuates pressure overload-induced cardiac remodeling and adverse cytoskeletal remodeling. **a**, **b** Histological analyses by PSR (**a**) staining showing increased myocardial fibrosis (**b**) in WT mice which were attenuated in GSNKO mice. **c**, **d** Taqman real-time PCR analyses showing increased mRNA expression of pro-collagen-I α1 (**c**) and pro-collagen-III α1 (**d**) in WT mice in response to pressure overload-induced biomechanical stress. Loss of gelsolin resulted in attenuation of pressure overload-induced mRNA expression of these extracellular matrix proteins. **e**–**g** Taqman real-time PCR analyses showing attenuation of pressure overload-induced increase in mRNA expression of cardiac disease markers including ANF (**e**), BNP (**f**), and β-MHC (**g**) in GSNKO mice compared with the WT mice at 9 and 18 weeks postsurgery. **h**–**j** Single cardiomyocyte contractility measurements (**h**) showing attenuation of decreased myofilament FS (**i**) and ±d*L*/d*t* (**j**) in cardiomyocytes isolated from GSNKO LVs compared with WT LVs in response to pressure overload for 9 weeks. **k**–**m** Representative images of F- and G-actin staining (**k**), F- to G-actin ratio (**l**), and actin-depolymerizing activity (**m**) showing increased actin depolymerization in WT hearts in response to pressure overload, whereas loss of gelsolin resulted in attenuation of actin-depolymerizing activity leading to increased F- to G-actin ratio. **n** Immunoprecipitation of gelsolin from WT—9 weeks TAC heart tissue homogenate resulting in marked attenuation of actin-depolymerizing activity. **o**–**q** Representative phase-contrast images (**o**) and quantification of viable cardiomyocytes (**p**) showing increased viability in GSNKO cardiomyocytes in response to 24-h cyclical stretch. Loss of gelsolin also resulted in greater increase F to G-actin ratio in response to 24-h cyclical stretch (**q**). In input, S supernatant from immunoprecipitate experiment. Data represent means ± s.e.m. ^*^P < 0.05 compared with the respective sham group, ^#^*P* < 0.05 compared with corresponding WT—9 Wk or 18 Wk TAC group as determined by unpaired two-tailed Student’s *t* test (**n**) and one-way ANOVA analysis (**b**–**g**, **i**, **j**, **l**, **m**, **p**, **q**). Biological replicates: *n* = 4 (**a**, **b**), *n* = 10 (**c**–**g**), *n* = 6 (**h**–**j**), *n* = 4 (**k**–**m**), and *n* = 3 (**o**–**q**). For in vitro experiments, each biological replicate was mean of technical replicates (**o**–**q**); only biological replicates are plotted and used for statistics. Scale bars show 25 µm (**a**, **k**) and 100 µm (**o**)

Deletion of gelsolin clearly mitigated pressure-overload induced pathological cardiac remodeling. Myocardial fibrosis and pro-fibrotic gene expression were reduced (Fig. 2a–d, Supplementary Fig. 4a, b and Supplementary Data 1), and α-smooth muscle actin (α-SMA) levels were attenuated (Supplementary Fig. 4c and Supplementary Data 1) suggesting reduced activation of fibroblast in gelsolin-deficient hearts in response to pressure overload. Myocardial hypertrophy and fetal gene reprogramming in response to pressure overload-induced biomechanical stress (Fig. 2e–g, Supplementary Fig. 4d and Supplementary Data 1) were also mitigated in gelsolin-deficient hearts. In response to pressure-overload, WT cardiomyocytes showed decreased contractility and relaxation (Fig. 2h–j and Supplementary Data 1), associated with decreased F/G-actin ratio (Fig. 2k, l and Supplementary Data 1) and increased actin-depolymerizing activity (Fig. 2m and Supplementary Data 1). In contrast, loss of gelsolin preserved cardiomyocyte function and F/G-actin ratio, consistent with a lack of increase in actin-depolymerizing activity (Fig. 2k–m). The preserved cytoskeletal architecture seen in pressure-overloaded GSNKO hearts was associated with dampened upregulation of the N-cadherin and β-catenin proteins at the intercalated discs (Supplementary Fig. 5 and Supplementary Data 1), reflecting enhanced adaptive mechanotransduction (Supplementary Fig. 5 and Supplementary Data 1). Affinity purified total proteins (after gelsolin immunoprecipitation) from pressure-overloaded WT hearts showed a marked reduction of actin-depolymerizing activity (Fig. 2n and Supplementary Data 1), documenting that gelsolin is a dominant actin-depolymerizing protein in the heart. To understand the role of cardiomyocyte-specific gelsolin in cardiac remodeling, we isolated and stretched adult cardiomyocytes from WT and GSNKO hearts. Gelsolin-null cardiomyocytes showed greater viability after 24 h stretch that was associated with a greater increase in actin polymerization (Fig. 2o–q and Supplementary Data 1). Taken together, our data demonstrate that deletion of gelsolin protects from advanced HF and uncover a critical role of adverse actin cytoskeletal remodeling in the pathogenesis of HF.

### Modeling interaction between gelsolin and phosphoinositides

**Fig. 3:**
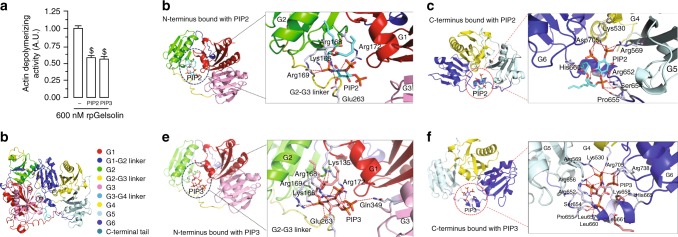
PIP2 and PIP3 bind with and inhibit gelsolin. **a** Actin-depolymerization assay showing identical inhibition of gelsolin by equimolar PIP2 and PIP3 in an in vitro lysate-free assay. **b** Complete model of human gelsolin structure illustrating its 6 domains (G1-G6) and the C-terminal tail. **c**, **d** Molecular modeling illustrating potential sites of interaction of the N-terminus (**c**) and C-terminus (**d**) of gelsolin with PIP2. **e**, **f** Molecular modeling illustrating potential sites of interaction of the N-terminus (**e**) and C-terminus (**f**) of gelsolin with PIP3. Please also see Supplementary Movies 1–4. Data represent means ± s.e.m. ^$^*P* < 0.05 compared with the 600 nM rpGelsolin group as determined by one-way ANOVA analysis (**a**). Biological replicates: *n* = 9 (**a**)

To assess the effects of PIP2 and PIP3, substrate and product of the PI3Kα catalytic activity, respectively, on the gelsolin actin-depolymerizing activity, we performed a lysate-free actin-depolymerization assay. Interestingly, equimolar PIP2 and PIP3 showed identical inhibition of gelsolin in a lysate-free assay (Fig. 3a and Supplementary Data 1). Gelsolin is composed of six domains, designated (from the N-terminus) as G1–G6 (Fig. 3b), and contains multiple phosphatidylinositol binding sites^19,20^. In silico modeling of gelsolin–PIP2 complex using comparative homology approach suggested that for human gelsolin, PIP2 binds with Lys166, Arg168, Arg169, Arg172 in the G1, G2 sub-domains, Glu263 in the G2–G3 linker in the N-terminal domain, and with residues in three C-terminal sub-domains, G4, G5, and G6 (Fig. 3c, d). Comparison of the binding and molecular interactions of the N- and C-terminal domains of human gelsolin with the PIP2 and PIP3 lipids, suggested that, compared to PIP2, PIP3 may have more binding partners in both the N- and C-terminal domains of gelsolin. Indeed, there are five additional H-bond interactions in the PIP3-bound N-terminus of gelsolin compared to the PIP2-bound complex, with PIP3 adopting 12 extra H-bonds compared to PIP2 in the C-terminal domain (Supplementary Table 2). Importantly, PIP3 showed multiple unique additional interactions including the interaction of the 3′ phosphate group of the inositol ring with Gln349 in the G3 sub-domain and the nonpolar aliphatic Leu657, and three H-bonds with Asp705 (Fig. 3e, f; Supplementary Table 2).

We next compared the molecular interaction of PIP2 and PIP3 with the N- and C-terminal domains of gelsolin using the trajectory obtained from molecular dynamics simulation (Supplementary Table 3). When PIP2 is bound to the gelsolin N- and C-terminal domains, the length of the various H-bonds fluctuates rapidly throughout the simulation, indicative of their instability (Supplementary Movies 1–2). In contrast, when PIP3 is complexed with gelsolin, there are a greater number of stable hydrogen bonds: an oxygen atom from one of the terminal phosphates of PIP3 forms two hydrogen bonds with Gln322 in the N-terminus of gelsolin, which remained stable throughout the trajectory (Supplementary Movie 3); C-terminal residues such as Arg131 and Lys223 form stable hydrogen bonds with PIP3 throughout the simulation (Supplementary Movie 4). The relative dynamics of PIP2 and PIP3 binding illustrate that PIP2 binds slightly towards the protein surface compared to PIP3, which instead remains bound within a region surrounded compactly with a greater number of residues in both the N- and C-terminal domains of gelsolin (Supplementary Movies 1–4).

### Biochemical and cellular effects of PIP3

**Fig. 4:**
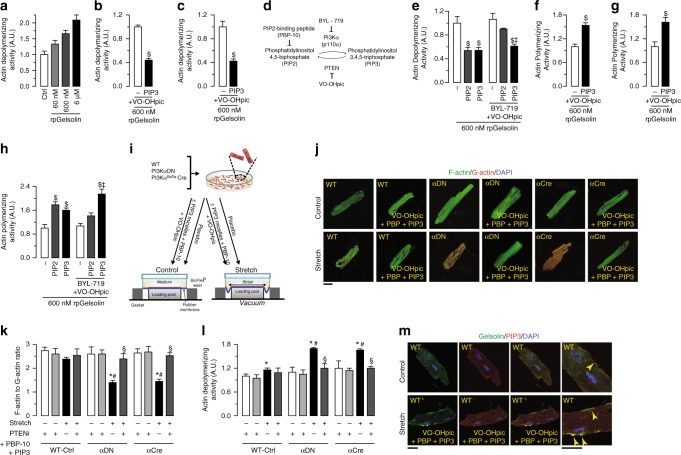
PI3Kα-generated PIP3 is a major negative regulator of gelsolin activity. **a** Concentration-dependent effect of rpGSN in actin-depolymerization assay. **b**, **c** Inhibition of actin-depolymerizing activity of 600 nM rpGSN (**b**) and 60 nM rhGSN (**c**) by PIP3 in presence of VO-OHpic. **d** A schematic showing the interconversion pathway of PIP2 and PIP3 by p110α and PTEN. **e** PIP2-mediated inhibition of exogenous gelsolin actin-depolymerizing activity in GSNKO heart tissue homogenate is partially blocked by BYL-719 and VO-OHpic, suggesting that the conversion to PIP3 is necessary for PIP2-mediated inhibition of gelsolin activity in ex vivo actin-depolymerization assay. **f**, **g** Actin polymerization assay showing inhibition of actin-capping activity of rpGSN (**f**) and rhGSN (**g**) by PIP3 in presence of VO-OHpic. **h** PIP2-mediated inhibition of exogenous gelsolin actin-capping activity in GSNKO heart tissue homogenate is partially blocked by BYL-719 and VO-OHpic, suggesting that the conversion to PIP3 is necessary for the PIP2-mediated inhibition of gelsolin actin-capping activity in ex vivo actin polymerization assay. **i** A schematic showing the experimental plan for isolated cardiomyocyte stretching in the absence or presence of the PIP3 micelles. **j**–**l** Representative images of F- and G-actin staining (**j**), F- to G-actin ratio (**k**), and actin-depolymerizing activity (**l**) showing increased actin depolymerization in the p110α transgenic cardiomyocytes subjected to cyclical stretch for 24 h. Addition of PIP3 micelles in presence of PBP-10 and VO-OHpic attenuated the increased actin-depolymerizing activity in stretched p110α transgenic cardiomyocytes. **m** Representative gelsolin and PIP3 IF staining images showing increased PIP3 levels in PIP3 micelles-treated cells. Arrows indicate spatial colocalization of gelsolin and PIP3. Data represent means ± s.e.m. ^$^*P* < 0.05 compared with respective controls, ^‡^*P* < 0.05 compared with PIP2 + BYL-719 + VO-OHpic group, ^*^P < 0.05 compared with nonstretch group, ^#^*P* < 0.05 compared with WT-Ctrl—Stretch group, ^§^*P* < 0.05 compared with respective Stretch group as determined by unpaired two-tailed Student’s *t* test (**b**, **c**, **f**, **g**) and one-way ANOVA analysis (**e**, **h**, **k**, **l**). Biological replicates: *n* = 6 (**a**–**c**, **f**–**h**, **j**–**m**) and *n* = 9 (**e**). For in vitro experiments, each biological replicate was mean of technical replicates (**j**–**m**); only biological replicates are plotted and used for statistics. Scale bars show 25 µm (**j**, **m**)

We next studied the biochemical effects of PIP3 and PIP2 on gelsolin’s depolymerizing activity on actin. We carried out a concentration-dependent actin-depolymerizing activity by recombinant porcine gelsolin (rpGSN) confirming the utility of the in vitro assay system (Fig. 4a, Supplementary Fig. 6a and Supplementary Data 1). In an actin-depolymerizing assay using GSNKO myocardial tissue lysate, spiked with recombinant porcine or human gelsolin (rhGSN), both PIP3 and PIP2 induced a marked suppression of actin-depolymerizing activity (Fig. 4b, c, Supplementary Fig. 6b and Supplementary Data 1). While the presence of a PTEN inhibitor preserved PIP3 ability to inhibit gelsolin activity (Fig. 4b, c, Supplementary Fig. 6b and Supplementary Data 1), pre-incubation of the myocardial tissue lysate with the PI3Kα-specific inhibitor, BYL-719, blocked 80% of the PIP2-mediated inhibition of gelsolin actin-depolymerizing activity, without affecting the effects of PIP3 (Fig. 4d, e). These data show that PI3Kα-mediated generation of PIP3 is essential for inhibition of gelsolin by PIP2 in myocardial tissue. Since gelsolin also potently caps actin filaments, we next assessed the actin polymerization using GSNKO myocardial tissue lysate, spiked with gelsolin. This assay showed increased actin polymerization (Fig. 4f, g and Supplementary Data 1), suggesting inhibition of gelsolin actin-capping activity by PIP3 in the presence of a PTEN inhibitor. As was observed with the actin-depolymerizing activity of gelsolin, pre-incubation of the tissue lysate with BYL-719, partially blocked the PIP2-mediated inhibition of gelsolin actin-capping activity, without affecting the effects of PIP3 (Fig. 4h and Supplementary Data 1).

To elucidate the cellular effects of PIP3 on gelsolin activity, we isolated adult cardiomyocytes and subjected them to cyclical stretch-induced biomechanical stress (Fig. 4i). We assessed the structural arrangements of cytoskeletal actin filaments using F-actin and G-actin double staining measured by confocal microscopy as an index of relative actin polymerization levels^21,22^. Biomechanical stress increased actin-depolymerizing activity and reduced the F/G-actin ratios in cardiomyocytes isolated from two different genetic murine models with reduced PI3Kα activity (PI3KαDN (αDN) and PI3Kα^flx/flx^ α-MHC-Cre (αCre)) as compared to WT controls (Fig. 4j–l and Supplementary Data 1). The addition of PIP3 micelles to these cells, along with PBP-10, a PIP2-binding peptide which sequesters PIP2, resulted in increased intracellular PIP3 levels, as assessed by immunofluorescence (IF) staining using a specific antibody to PIP3 (Fig. 4m). Importantly, this PIP3 prevented these inhibitory effects of p110α inactivation, indicative of a key role of PI3Kα (p110α)-generated PIP3 in the regulation of cytoskeletal remodeling in response to biomechanical stress (Fig. 4j–l and Supplementary Data 1). Importantly, IF staining also showed spatial colocalization between gelsolin and PIP3, which was predominantly at the cell periphery (Fig. 4m).

**Fig. 5:**
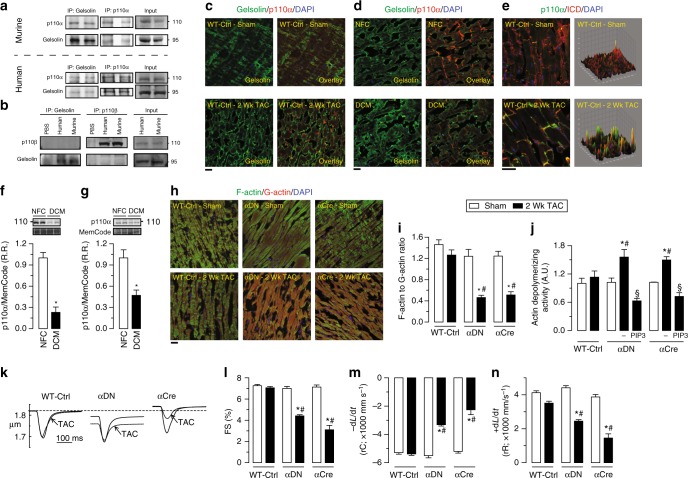
p110α, but not p110β, interacts with gelsolin. **a** Co-immunoprecipitation and immunoblotting showing that p110α and gelsolin interact in murine and human hearts. **b** Co-immunoprecipitation and immunoblotting showing no interaction between p110β and gelsolin in murine and human hearts. **c**, **d** Representative IF staining images showing spatial colocalization between p110α and gelsolin in murine (**c**) and human (**d**) hearts. **e** Representative IF staining images for p110α and N-cadherin in murine hearts showing the localization of p110α at the intercalated discs. Surface plots were plotted using Fiji ImageJ (NIH) show a graphical representation of intensity profiles of the IF images. **f**–**g** Western blot analyses showing decreased myocardial levels of p110α in human (**f**) and canine (**g**) hearts with DCM compared with the NFC donor hearts. **h**–**j** Representative images of F- and G-actin staining (**h**), F- to G-actin ratio (**i**), and actin-depolymerizing activity derived from actin-depolymerization assay (**j**) showing a greater degree of actin depolymerization in p110α transgenic mice in response to pressure overload-induced biomechanical stress. Addition of PIP3, in the presence of VO-OHpic, inhibited the actin-depolymerizing activity (**j**). **k**–**n** Single cardiomyocyte contractility measurements (**k**) showing decreased myofilament FS (**m**) and ±d*L*/d*t* (**l**–**n**) in LV cardiomyocytes isolated from 2 weeks of pressure-overloaded p110α transgenic mice hearts. Data represent means ± s.e.m. ^*^P < 0.05 compared with the respective sham/NFC groups, ^#^*P* < 0.05 compared with WT-Ctrl—2 week TAC group as determined by unpaired two-tailed Student’s *t* test (**f**, **g**) and one-way ANOVA analysis (**i**, **j**, **l**–**n**). Biological replicates: *n* = 4 (**a**–**j**) and *n* = 6 (**k**–**n**). Scale bars show 25 µm (**c**, **d**, **e**, **h**)

Immunoprecipitates of gelsolin from murine and human myocardial tissue contained immunoreactivity for p110α, but not for p110β, the other broadly expressed class I PI3K catalytic subunit (Fig. 5a, b). Reciprocal co-immunoprecipitation using antibodies against p110α or p110β confirmed an interaction between gelsolin and p110α, but not p110β (Fig. 5a, b). Double IF staining for p110α and gelsolin in murine and human hearts confirmed a spatial colocalization between these proteins (Fig. 5c, d and Supplementary Fig. 7). Following pressure-overload, p110α showed increased translocation to the intercalated discs, key subcellular areas involved in sensing biomechanical stress in the heart (Fig. 5e). Taken together, these results highlight a key regulatory role of the PI3Kα-gelsolin complex in mechanotransduction, with the marked decrease in p110α levels in human and canine DCM hearts further suggesting a causal role in HF most likely due to a reduced PIP3-mediated suppression of gelsolin activity (Fig. 5f, g and Supplementary Data 1).

We next characterized two different transgenic mice selectively lacking p110α activity in cardiomyocytes (αDN and αCre), and WT controls (WT-Ctrl) using a model of pressure-overload mediated HF (Supplementary Fig. 2B). WT hearts displayed an intact arrangement of intracellular actin filaments, but the filaments were largely disorganized and interrupted in pressure-overloaded PI3Kα mutant hearts (Fig. 5h, i and Supplementary Data 1), correlating with a marked increase in actin-depolymerizing activity which could be suppressed by the addition of PIP3 (Fig. 5j and Supplementary Data 1). In contrast, pressure-overloaded PI3Kα mutant hearts showed disrupted intracellular filamentous actin, with decreased cardiomyocyte contractility and relaxation compared to WT hearts (Fig. 5k–n and Supplementary Data 1). Importantly, loss of p110α only affected the cytoskeletal F-actin (microfilaments) and not the sarcomeric thin filaments as assessed by α-sarcomeric actin staining (Supplementary Fig. 8a). In summary, loss of PI3Kα kinase activity in the heart markedly increased susceptibility to biomechanical stress leading to the disruption of the intracellular actin cytoskeleton and reduced cardiomyocyte contractility, which could be restored by the addition of PIP3, the lipid product of PI3Kα.

### Loss of PI3Kα leads to cytoskeletal remodeling and HF

**Fig. 6:**
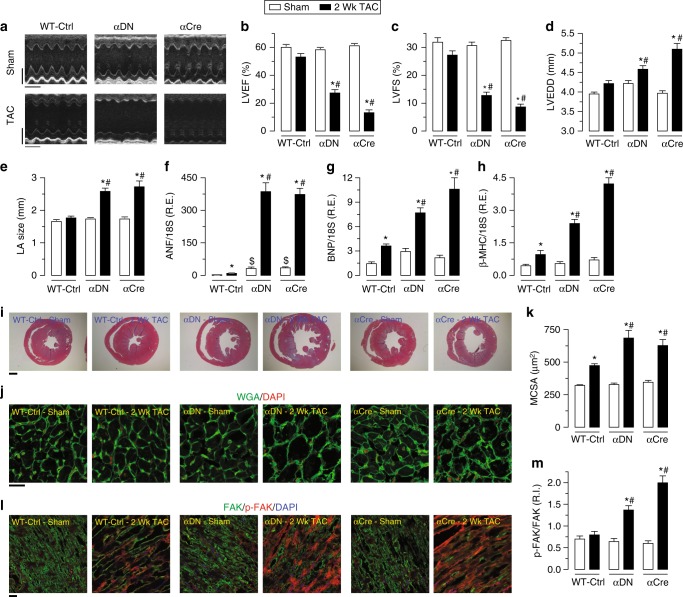
Loss of p110α leads to accelerated HF in response to pressure overload. **a**–**e** Representative M-mode echocardiography images of LV (**a**) and quantification of cardiac function showing severely decreased LVEF (**b**) and LVFS (**c**) along with increased LV end-diastolic dimension (LVEDD; **d**) and left atrium (LA) size (**e**) in p110α transgenic mice, αDN (PI3KαDN) and αCre (PI3Kα^flx/flx^ Cre), in response to pressure overload-induced biomechanical stress, compared with preserved cardiac function in the WT mice. **f**–**h** Taqman real-time PCR analyses showing a greater increase in mRNA expression of cardiac disease markers including ANF (**f**), BNP (**g**), and β-MHC (**h**) in p110α transgenic mice compared with the WT mice in response to pressure overload for 2 weeks. **i**–**k** Histological analyses by Masson trichrome staining (**i**) and WGA staining (**j**) showing a greater increase in ventricular dilation (**i**), cardiac fibrosis (**j**), and myocyte cross-sectional area (**j**, **k**) in p110α transgenic mice compared with the WT mice in response to pressure overload. **l**, **m** Representative IF staining images showing unchanged phosphorylation of FAK in WT LVs, in contrast to significantly increased phosphorylation of FAK in p110α transgenic LVs in response to pressure overload (Fig. 3d–f). Data represent means ± s.e.m. ^*^P < 0.05 compared with the respective Sham groups, ^#^*P* < 0.05 compared with WT-Ctrl—2 Wk TAC group as determined by one-way ANOVA analysis (**b**–**h**, **k**, **m**). Biological replicates: *n* = 12 (**a**–**e**), *n* = 10 (**f**–**h**) and *n* = 4 (**i**–**m**). Scale bars show 2 mm (*y*-axis of **a**), 200 ms (*x*-axis of **a**), 1 mm (**i**), and 25 µm (**j**, **l**)

We next examined the HF phenotype in PI3Kα-deficient mice in response to pressure-overload and further characterized the involvement of gelsolin and its interaction with p110α function. Loss of PI3Kα lipid kinase activity in the heart resulted in increased susceptibility to HF with an accelerated development of a severe DCM (Fig. 6a–e and Supplementary Data 1). The exacerbated HF phenotype in pressure-overloaded PI3Kα mutant hearts was characterized by increased fetal gene reprogramming (Fig. 6f–h and Supplementary Data 1), increased cardiomyocyte cross-sectional area and ventricular dilation (Fig. 6i–k and Supplementary Data 1) coupled with increased myocardial fibrosis (Supplementary Fig. 8b–d and Supplementary Data 1). The intercellular N-cadherin/β-catenin complex^9,23,24^ and the integrin-based/focal adhesion kinase (FAK) complex are important mechanosensors^25,26^. In response to pressure-overload, upregulation of these sensors was enhanced in PI3Kα mutants compared with WT hearts (Fig. 6l–m, Supplementary Fig. 9 and Supplementary Data 1). At baseline, PI3Kα mutants were not different in other key mediators of myocardial remodeling such as the phosphorylation of Akt (T308) and phospholamban (Ser16/Thr17), the levels of sarco(endo)plasmic reticulum Ca^2+^-ATPase (SERCA2a) and calpain, L-type Ca^2+^ current (I_Ca,L_), and the degree of apoptosis between WT and PI3Kα mutant hearts (Supplementary Fig. 10 and Supplementary Data 1). Moreover, p110β^flx/flx^ Cre (βCre) and p110β^flx/flx^ mice subjected to pressure-overload induced biomechanical stress showed similar cardiac hypertrophy and fetal gene reprogramming as WT hearts (Supplementary Fig. 11 and Supplementary Data 1), with intact F-actin, F/G-actin ratio and actin-depolymerizing activity (Supplementary Fig. 12 and Supplementary Data 1). These data establish PI3Kα specificity in the adverse cytoskeletal remodeling in response to biomechanical stress.

**Fig. 7:**
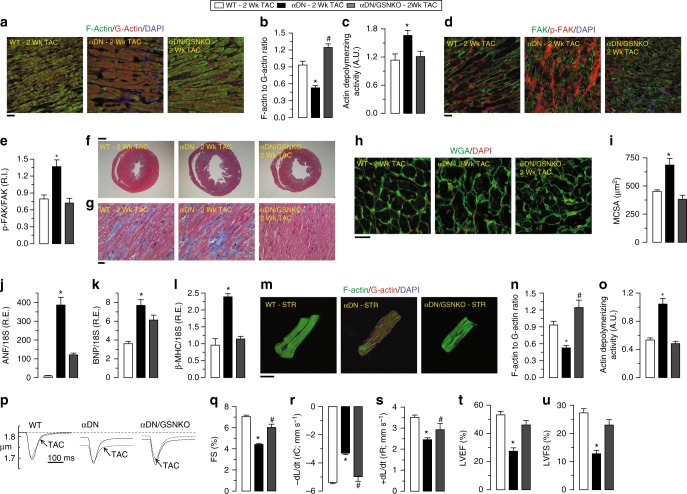
Loss of gelsolin attenuates the biomechanical stress-induced cytoskeletal remodeling and preserves the cardiac function in PI3KαDN mice. **a**–**c** Representative images of F- and G-actin staining (**a**), F- to G-actin ratio (**b**), and actin-depolymerizing activity (**c**) showing pressure overload-induced dysregulation of actin filaments (reduction of F/G-actin ratio) and increased actin-depolymerizing activity in the PI3KαDN LVs. Loss of gelsolin (in αDN/GSNKO mice) resulted in reduced actin-depolymerizing activity and preserved actin filament arrangement (preserved F/G-actin ratio) in response to pressure-overload. **d**, **e** Representative IF images (**d**) and their quantification (**e**) showing attenuated phosphorylation of FAK in αDN/GSNKO hearts in response to pressure overload. **f**–**i** Masson trichrome (**f**, **g**) and WGA staining (**h**) showing alleviation of ventricular dilation (**f**), cardiac fibrosis (**g**), and myocyte cross-sectional area (**h**, **i**) in αDN/GSNKO mice compared with the αDN mice in response to pressure overload. **j–l** Taqman real-time PCR analyses showing attenuation of increased mRNA expression of cardiac disease markers including ANF (**j**), BNP (**k**), and β-MHC (**l**) in αDN/GSNKO mice compared with the αDN mice in response to pressure overload-induced biomechanical stress. **m**–**o** Representative images of F- and G-actin staining (**m**), F- to G-actin ratio (**n**), and actin-depolymerizing activity (**o**) showing the attenuation of increased actin-depolymerizing activity in the cardiomyocytes isolated from αDN/GSNKO LVs compared with αDN LVs subjected to cyclic stretch for 24 h. **p**–**s** Single cardiomyocyte contractility measurements (**p**) showing attenuation of decreased myofilament FS (**q**) and ±d*L*/d*t* (**r**, **s**) in cardiomyocytes isolated from αDN/GSNKO LVs compared with αDN LVs in response to pressure overload. **t**–**u** Quantitative assessment of M-mode echocardiography of LV showing preserved cardiac function in αDN/GSNKO hearts in response to pressure overload-induced biomechanical stress. Data represent means ± s.e.m. ^*^P < 0.05 compared to all the groups, ^#^*P* < 0.05 compared with αDN—2 week TAC group as determined by one-way ANOVA analysis (**b**, **c**, **e**, **i**–**l**, **n**, **o**, **q**–**u**). Biological replicates: *n* = 4 (**a**, **b**, **d**–**i**), *n* = 6 (**c**, **m**–**s**), *n* = 10 (**j**–**l**) and *n* = 12 (**t**, **u**). For in vitro experiments, each biological replicate was mean of four technical replicates (**m**–**o**); only biological replicates are plotted and used for statistics. Scale bars show 25 µm (**a**, **d**, **g**, **h**, **m**) and 1 mm (**f**)

To test the role of gelsolin in mediating heart disease in the setting of reduced PI3Kα function, we next generated double-mutant mice by intercrossing the dominant-negative PI3Kα (PI3KαDN) mice with GSNKO mice, generating PI3Kα dominant-negative GSNKO double-mutant (PI3KαDN/GSNKO) mice (Supplementary Fig. 2b). In contrast to PI3KαDN hearts, PI3KαDN/GSNKO hearts showed preserved F/G-actin ratio and actin-depolymerizing activity in response to pressure-overload induced biomechanical stress (Fig. 7a–c, Supplementary Fig. 13a and Supplementary Data 1). Preservation of the actin cytoskeleton in PI3KαDN/GSNKO hearts resulted in normalization of protein levels of N-cadherin, β-catenin, and phosphorylation of FAK in response to pressure-overload (Fig. 7d, e, Supplementary Fig. 14 and Supplementary Data 1). Importantly, double-mutant hearts showed attenuated pathological cardiac remodeling with reduced ventricular dilation and myocardial fibrosis (Fig. 7f, g, Supplementary Fig. 13b, c and Supplementary Data 1), hypertrophy (Fig. 7h, i and Supplementary Data 1) and fetal gene reprogramming (Fig. 7j–l and Supplementary Data 1). Importantly, loss of gelsolin also attenuated cyclic stretch-induced biomechanical stress-mediated adverse cytoskeletal remodeling in isolated cardiomyocytes (Fig. 7m–o and Supplementary Data 1) which is reflected in maintained cardiomyocyte contractility and relaxation from pressure-overloaded PI3KαDN/GSNKO hearts (Fig. 7p–s and Supplementary Data 1). Echocardiographic assessment in response to 2 weeks of pressure-overload revealed that loss of gelsolin in PI3KαDN/GSNKO hearts prevented ventricular dilation and preserved cardiac function (Fig. 7t, u, Supplementary Figs. 13d, e and 14, and Supplementary Data 1). These results provide clear genetic evidence that PI3Kα drives gelsolin-mediated adverse cytoskeletal remodeling in response to biomechanical stress.

## Discussion

**Fig. 8:**
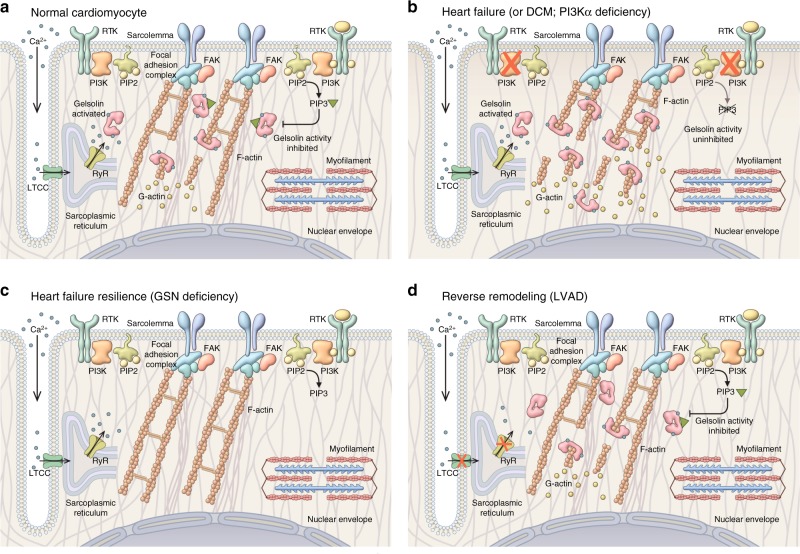
Regulation of cytoskeleton density by PI3Kα. **a** Normal myocyte: active PI3Kα produces a pool of PIP3 that suppresses excessive activation of gelsolin (GSN) by Ca^2+^ during Ca^2+^ cycling leading to moderate gelsolin severing activity, normal cytoskeleton (F-actin) density, and good resilience to biomechanical stress. **b** Heart failure (e.g., dilated cardiomyopathy, DCM) or PI3Kα-deficient model under pressure overload: low-levels or absent PI3Kα activity leads to low levels of PIP3. Lack of PIP3 result in unhindered (high) gelsolin activation during Ca^2+^ cycling, excessive breakdown of cytoskeleton (F-actin), low-cytoskeleton density, and poor resistance to biomechanical stress leading to DCM. **c** Heart failure resilience due to GSN deficiency: in the absence of gelsolin (GSN) and actin-severing activity associated with it, myocytes are able to maintain a high density of cytoskeleton (F-actin) resulting in high resilience to biomechanical stress and linked heart failure. **d** Reverse remodeling (LVAD): in the presence of left-ventricular assist device, heart contraction and associated Ca^2+^ release are of much lower magnitude. Low levels of Ca^2+^ during Ca^2+^ cycling (release) result in less Ca^2+^-activation of gelsolin (inactive gelsolin) moderating gelsolin actin-severing activity that leads to improvement in cytoskeletal (F-actin) density, which in turn may drive reverse remodeling

Mechanotransduction, the conversion of biomechanical stimuli into signal transduction, is mediated by interactions between the intracellular cytoskeletal network with intercellular (cell–cell) and extracellular (cell–extracellular matrix) complexes (Fig. 8)^6,9^. In the heart, these complexes include the N-cadherin and β-catenin complexes in intercalated discs^4,24,27^ and integrin-mediated recruitment and auto-phosphorylation of FAK at the cell–extracellular matrix junctions^28^. The heart is an organ with a high requirement for precise mechanotransduction and remodeling of the actin cytoskeleton, as illustrated by loss-of-function mutations in cytoskeletal proteins, is associated with the progression of DCM and HF in humans^29–31^. In particular, cardiac mechanotransduction plays a fundamental role in response to biomechanical stress as observed in patients with hypertension^5,6,9^.

The reduced p110α levels in advanced HF and PI3Kα-deficient animal models (αDN and αCre) lowers PIP3 production in response to biomechanical stress. Lack of suppression of gelsolin activity by PIP3 leads to the excessive breakdown of cytoskeleton compromising the structural integrity of cardiomyocytes (Fig. 8b). Disrupted cytoskeleton can also lead to secondary changes including dysregulation of L-type Ca^2+^ current^32^ compromising excitation–contraction coupling and contributing to contractile dysfunction. A murine model with cardiomyocyte-specific loss of PTEN leads to constitutively high PI3Kα activity, and PIP3 levels were protected from pressure overload mediated HF^10,18^ further confirming an important role of PI3Kα/PIP3 axis in protecting against biomechanical stress. Besides PI3Kα, closely related isoform PI3Kβ is also present in the heart^11,33^, but is not involved in pressure-overload related remodeling since there was no difference in response to pressure overload between hearts with cardiomyocyte-specific deletion of p110β (βCre) and their littermates with intact p110β. Loss of gelsolin in the PI3KαDN background largely prevented adverse cytoskeletal remodeling and HF underlying the importance of the cytoskeleton in the progression of pressure-overload induced HF. Gelsolin is a broadly expressed Ca^2+^-regulated actin filament severing and capping protein^7,20^ known to regulate cell motility^34^. In the absence of gelsolin, pressure overload cannot trigger cytoskeleton breakdown (Fig. 8c) preserving myocytes structural integrity and contractility (no excitation–contraction coupling disruption due to cytoskeleton-related disruption of I_Ca,L_). GSNKO mice exhibited no baseline cardiovascular defects, suggesting that multiple other actin-severing proteins are likely to compensate for the basal loss gelsolin^35^ and indicating that gelsolin is selectively involved in the progression of pressure-overload mediated HF. Moreover, in human samples from DCM hearts, gelsolin activity is correlated with severity of myocardial dysfunction corroborating an important role gelsolin plays in the progression of DCM; therefore, pharmacological inhibition of gelsolin is a promising therapeutic approach to prevent adverse cytoskeletal remodeling in DCM. Adverse myocardial remodeling is a complex process of cardiomyocyte hypertrophy, fibrosis, and energetics coupled with altered signaling^27,36,37^. Mechanical devices, such as LVAD, result in immediate pressure and volume unloading of the LV^38,39^. We found that failing human hearts with DCM and elevated gelsolin actin-depolymerizing activity responded to LVAD therapy by a marked improvement in cytoskeletal integrity possibly due to reduced Ca^2+^ influx reducing Ca^2+^-dependent activation of gelsolin and its severing activity thus improving cytoskeletal integrity (Fig. 8d). These results further strengthen the clinical utility of LVAD therapy and suggest a novel mechanism of action.

Importantly, we established that gelsolin is a major determinant in biomechanical stress-mediated advanced HF evidenced by improved survival, preserved systolic function, and molecular, cellular, and histological alterations of pressure-overloaded gelsolin mutant mice compared to littermate WT controls. However, since gelsolin knockout was not limited to cardiomyocytes, other cell types, including cardiac fibroblasts, could have contributed to the protection from pressure overload. Gelsolin is also highly abundant in fibroblasts where it is responsible for the actin filament organization^7,40^, regulation of α-SMA expression^41^ and their transformation into myofibroblasts. Although our data suggest a key regulation of gelsolin activity by PI3Kα-generated PIP3 in cardiomyocytes, this phenomenon may also exist in cardiac fibroblasts and may have contributed to the reduced myocardial fibrosis seen in the pressure-overloaded GSNKO mice. While decreased cytoskeletal remodeling in cardiomyocytes in vivo might have contributed to decreased myocardial fibrosis due to less mechanical stress, the role of gelsolin-mediated cytoskeletal remodeling in cardiac fibroblast and its implications in DCM warrant further investigation. Interestingly, gelsolin is also present in close proximity to sarcomeric actin, in addition to F-actin in microfilaments. However, thin filaments in cross-striated myofibrils in skeletal muscles are resistant to the severing action of gelsolin due to the presence of nebulin^42^. The cardiac-specific nebulin isoform, called nebulette, confer gelsolin resistance to the sarcomeric actin filaments in the heart, and we did not observe disruptions in α-sarcomeric actin, confirming the selective role of gelsolin in cytoskeletal microfilaments actin severing. Furthermore, loss-of-function mutations in nebulette are associated with DCM linked to the disrupted cytoskeleton in cardiomyocytes^43^.

In this study, we identified a critical mechanism by which the adaptive function of PI3Kα acts through the generation of PIP3 and suppression of gelsolin activity mitigating adverse remodeling of the intracellular actin cytoskeleton in cardiomyocytes using explanted human hearts, cardiomyocyte-specific transgenic mice, and lysate-based actin-depolymerizing activity assay (Fig. 8). We found identical inhibition of gelsolin activity by equimolar PIP2 and PIP3 using an in vitro lysate-free assay as reported previously^19,44^. In response to biomechanical stress, PI3Kα (p110α) translocates to the intercalated discs and plasma membrane, where PI3Kα converts PIP2 to PIP3. This PIP3 sequesters out gelsolin to the plasma membrane, displaying a spatial colocalization of p110α and gelsolin and provides a basis for a localized regulation of gelsolin activity by PI3Kα (but not PI3Kβ) in both human and murine hearts, where p110α-catalyzed PIP3 negatively regulates gelsolin activity thereby maintaining cytoskeletal integrity of cardiomyocytes (Fig. 8a). Additionally, PIP2 binds with multiple binding partners, including but not limited to cofilin, vinculin, moesin, spectrin, alpha-actinin, and various other proteins^45–48^ and this competitive binding of PIP2 may limit its bioavailability for gelsolin binding in vivo.

The capacity of PI3Kα inhibition to block PIP2-mediated inhibition of actin-depolymerizing and actin-capping activity in tissue lysate, suggests that the majority of PIP2 effects on actin-depolymerizing are mediated by PI3Kα-mediated conversion into PIP3. Our data demonstrate that PIP3 plays a key role in suppressing gelsolin-mediated actin-depolymerizing as well as capping of the barbed end of F-actin thereby allowing the elongation of F-actin. Similarly, our experiments with exogenous PIP3 were carried out in the presence of PTEN inhibition thereby preventing the generation of PIP2; these studies recapitulated the observations made in cardiomyocyte-specific mutant PTEN mice. Loss of cardiomyocyte PTEN enhances PI3Kα action^10,49^ thereby protecting the heart from biomechanical stress^18^. The other major PI3K isoform in the heart, PI3Kγ, also plays a key adaptive role in mechanotransduction. Loss of p110γ function results in elevated cAMP levels, upregulated matrix metalloproteinases, and degradation of N-cadherin leading to exacerbated pressure-overload mediated HF^4^. As such, the PI3K family controls mechanotransduction in the heart via distinct modes of regulation: PI3Kα, which is typically activated by tyrosine-receptor kinase agonizts, negatively regulates gelsolin activity and protects the intracellular cytoskeleton while PI3Kγ, which is activated by G-protein coupled receptors, negatively regulates cAMP and protects the N-cadherin cell adhesion complexes. Biomechanical stress at the intercellular junction, sensed by N-cadherin, promotes actin polymerization through regulation of gelsolin and actin assembly^50^, suggesting a possible cooperative relationship between distinct PI3K isoforms in heart disease.

## Methods

### Experimental animals and protocol

GSNKO mice were used^7^. PI3KαDN mice express a catalytically inactive p110α under the cardiac-specific α-MHC promoter^51^. These mice were crossed with GSNKO mice to generate PI3KαDN/GSNKO double-mutant mice. Mice with transgenic Cre recombinase under the control of the αMHC promoter (Jackson Laboratories, Bar Harbor, ME) were crossed with mice in which the sequences encoding the key parts of the catalytic kinase domain of p110α (*Pik3ca*) or p110β (*Pik3cb*) genes were flanked by *loxP* sites^52,53^. Littermate non-Cre and WT mice were used as pooled controls (Ctrl). All experiments were performed in accordance with Institutional guidelines, Canadian Council on Animal Care, and the Guide for the Care and Use of Laboratory Animals published by the US National Institutes of Health (revised 2011). All studies were approved by the Animal Care and Use Committee at the University of Alberta.

### Human and canine explanted hearts

Our study was approved by the Ethics Committee at the University of Alberta, and all patients provided written informed consents in accordance with the Declaration of Helsinki (2008) of the World Medical Association. LV tissues were harvested from explanted human failing hearts and donor nonfailing control (NFC) human hearts which were preserved in cold cardioplegia solution via Human Explanted Heart Program at the Mazankowski Alberta Heart Institute and the Human Organ Procurement and Exchange program at the University of Alberta Hospital, respectively, and rapidly snap-frozen in liquid nitrogen within 15 min of explantation. Canine myocardial samples were obtained from the LV free wall of dogs with advanced DCM resulting in HF whose owners elected humane death or dogs with no previous history of cardiovascular disease (NFCs)^54^. Samples were rapidly frozen in liquid nitrogen and stored at −80 °C. Written consent was obtained from all patients and clients.

### Transverse aortic constriction

Young (8–8½-week old) GSNKO, WT littermate controls, PI3KαDN (αDN), PI3Kα^flx/flx^ Cre (αCre), PI3Kβ^flx/flx^ Cre (βCre), and PI3KαDN/GSNKO (αDN/GSNKO) male mice were subjected to transverse aortic constriction (TAC)-induced pressure overload^4,5,18,55,56^. Sham-treated animals underwent the same procedure without the aortic constriction.

### Echocardiography and pressure–volume loop analyses

Transthoracic echocardiography and tissue Doppler imaging was performed noninvasively and analyzed in a blinded manner using a Vevo 3100 high-resolution imaging system equipped with a 30-MHz transducer (RMV-707B; VisualSonics, Toronto, Canada)^56,57^. LV pressure–volume analysis was performed using a 1.2F PV catheter (Scisense, Canada)^58,59^.

### Isolated cardiomyocyte contractility

Measurement of isolated cardiomyocyte contractility was performed as described^4^. Briefly, cardiomyocytes were perfused with modified Tyrode’s solution containing 1.2 mM Ca^2+^ at 35–36 °C and paced with field stimulation at 1 Hz. Sarcomere length was estimated in real time by software from images captured by the high-speed camera at a rate 200frames^−1^. Measurements of fractional shortening, and ±d*L*/d*t* were done at steady state (past 2 min of continuous stimulation). Only cardiomyocytes producing contraction of stable amplitude and kinetics at steady state were selected for analysis.

### Histology, wheat-germ and F-/G-actin staining, and IF

Hearts were arrested in diastole with 1 M KCl, fixed in 10% buffered formalin, and embedded in paraffin. Ten-micrometer-thick sections were stained with picrosirius red or Masson trichrome to assess myocardial fibrosis and were visualized using fluorescence microscopy (Olympus IX81) and light microscopy (DM4000 B, Leica), respectively, as described^56,57^. Five-micrometer-thick OCT-embedded cryosections were stained with Oregon Green 488-conjugated wheat-germ agglutinin (WGA; #W6748, ThermoFisher) and DAPI (#D3571, ThermoFisher) and visualized under a fluorescence microscope (Olympus IX81) to assess cardiomyocyte cross-sectional area^56^. For the α-sarcomeric actin staining, OCT-embedded heart cryosections were fixed in 4% paraformaldehyde and permeabilized in 100% methanol. After blocking, the sections were incubated with the α-sarcomeric actin antibody (#M0874, Dako; 1:50) followed by secondary antibody incubation, co-staining with Texas Red-X conjugated WGA (#W21405, ThermoFisher) and visualized using fluorescence microscopy (Olympus IX81).

Five-micrometers thick OCT-embedded cryosections and isolated cardiomyocytes were stained with Alexa Fluor 488-conjugated phalloidin (#A12379, ThermoFisher), Alexa Fluor 594-conjugated DNase I (#D12372, ThermoFisher), tetramethylrhodamine-WGA (#W849, ThermoFisher) and DAPI to visualize F-actin, G-actin, cell membranes, and nuclei, respectively. F-actin and G-actin staining intensities were quantified using Fiji ImageJ (NIH) from these images, and the F-actin to G-actin ratio was utilized as an index for actin polymerization. Tissue sections and isolated cells were visualized using confocal microscopy (Leica SP5, Leica Microsystems).

### Actin depolymerization and capping assays

A commercially available kit (Actin polymerization kit #BK003, Cytoskeleton Inc.) was used to assess actin-depolymerizing activity^19^. Briefly, “buffer A” was prepared by mixing general actin buffer (5 mM Tris-HCl pH 8.0 and 0.2 mM CaCl_2_; #BSA01-010, Cytoskeleton, Inc.) with ATP stock (100 mM; #BSA04-001, Cytoskeleton, Inc.) and actin polymerization buffer (500 mM KCl, 20 mM MgCl_2_, 0.05 M guanidine carbonate, and 10 mM ATP; #BSA02-001, Cytoskeleton, Inc.). The final composition of “buffer A” is 5 mM Tris-HCl pH 8.0, 0.2 mM CaCl2, 0.45 mM ATP, 12.5 mM KCl, 0.5 mM MgCl_2_, and 1.25 µM guanidine carbonate. The pyrene-labeled F-actin was prepared by incubating 0.4 mg ml^−1^ pyrene-labeled muscle actin (#AP05, Cytoskeleton, Inc.) with “buffer A” for 1 h at room temperature. Tissue and cellular proteins were prepared in phosphate buffered saline (137 mM NaCl, 2.7 mM KCl, 10 mM Na_2_HPO_4_ and 1.8 mM KH_2_PO_4_) pH 7.4 with 1× cOmplete Protease (#11697498001, Millipore Sigma) and PhosSTOP Phosphatase (#4906845001, Millipore Sigma) inhibitor cocktails.

To perform the actin-depolymerization assay, pyrene-labeled F-actin (substrate) was incubated with total proteins isolated from various tissues and cells (as described above), recombinant porcine cytosolic (#8304-1, Hypermol, UK) or recombinant human plasma gelsolin which was synthesized using the *Escherichia coli* expression system, purified and characterized^60,61^. The recombinant gelsolin was pre-incubated (20 min at room temperature) with PIP2 (#P-4508, Echelon Biosciences) or PIP3 (#P-3908, Echelon Biosciences) to assess their effects on actin depolymerization. The final composition in each assay well was 0.2 mg ml^−1^ pyrene-labeled F-actin, 100 µg of protein (isolated from tissues or cells), 2.5 mM Tris-HCl pH 8.0, 0.1 mM CaCl_2_, 0.225 mM ATP, 6.25 mM KCl, 0.25 mM MgCl_2_, and 0.625 µM guanidine carbonate with or without 20 µM PIP2 or PIP3.

In a lysate-free assay, 20 µM of PIP2 or PIP3 were pre-incubated (20 min) with rpGSN (600 nM), and their effect on the inhibition of gelsolin actin-depolymerization activity was assessed. The final composition in each assay well was 0.2 mg ml^−1^ pyrene-labeled F-actin, 2.5 mM Tris-HCl pH 8.0, 0.1 mM CaCl_2_, 0.225 mM ATP, 6.25 mM KCl, 0.25 mM MgCl_2_, and 0.625 µM guanidine carbonate with or without 20 µM PIP2 or PIP3. The actin-depolymerization assay was carried out as described above. Decay in the fluorescence was recorded using a microplate reader (Spectramax M5, Molecular Devices) and presented as actin-depolymerizing activity.

In a lysate-based assay gelsolin (600 nM of rpGSN and 60 nM of rhGSN) was spiked to the GSNKO cardiac whole cell lysate proteins (extracted from heart tissues as described above). Effects of equimolar (20 µM) PIP2 (#P-4508, Echelon Biosciences) and PIP3 (#P-3908, Echelon Biosciences) were evaluated on the gelsolin actin-depolymerization activity using the biochemical kit after 20 minutes pre-incubation of gelsolin with PIP2/PIP3. The actin-depolymerization assay was carried out as described above. Decay in the fluorescence was recorded using a microplate reader (Spectramax M5, Molecular Devices) and presented as actin-depolymerizing activity. In human DCM samples, the actin-depolymerizing assay was performed following immunoprecipitation of gelsolin.

Actin polymerization assays were conducted to assess the effect of PIP2 and PIP3 on actin-capping activity of recombinant porcine cytosolic and human plasma gelsolin. Briefly, G-actin (substrate) was prepared by reconstitution of 2 μM pyrene-labeled muscle (0.1 mg ml^−1^) actin in the “buffer A” as described above. To initiate actin-capping, we treated G-actin (2 μM) with “buffer A” (5 mM Tris-HCl pH 8.0, 0.2 mM CaCl2, 0.45 mM ATP, 12.5 mM KCl, 0.5 mM MgCl_2_, and 1.25 µM guanidine carbonate) and 100 µg total protein extracted from the GSNKO hearts (as described above). The increase in fluorescence was recorded overnight using the microplate reader (Spectramax M5) and was expressed as actin-polymerizing activity. Effects of recombinant porcine/human gelsolin and PIP2 or PIP3 (20 µM; Echelon Biosciences) were also recorded.

### TaqMan real-time PCR and Western blot analyses

Messenger RNA levels were quantified with TaqMan Real-Time PCR using ABI Prism 7700 sequence detection system as described previously^4,56^. A list of primers and probes along with their sequences are presented in Supplementary Table 4. Co-immunoprecipitation and Western blot analyses were performed as described^4,57^. Uncropped western blot images of data shown in Figs. 2 and 5 and Supplementary Figs. 1 and 10 can be found in Supplementary Fig. 15.

### Computer modeling and molecular dynamic simulation

Using the X-ray structure of human gelsolin^8^, comparative homology modeling was used to model the full-length structure of human gelsolin (782 amino acids) using this crystal structure (PDB ID: 3FFN) as a template (Modeller 9.14; http://salilab.org/modeller/)^62,63^. We studied comparative binding and molecular interactions between the N- and C-terminus domains of human gelsolin with PIP2 and PIP3^64,65^. Molecular dynamic simulations of the PIP2- and PIP3-bound gelsolin complexes were performed using GROMACS 5.1.2 software package^66^. Normal mode analysis^67,68^ and principal component analysis^69–71^ were used to model the dynamic changes in gelsolin structure in response to PIP2 and PIP3 binding.

### Isolation, culture, and stretching of adult cardiomyocytes

Adult murine LV cardiomyocytes were isolated from WT, GSNKO, αDN, αCre, and αDN/GSNKO mice, and cultured^5,57,72^. Cardiomyocytes were cyclically stretched at 1 Hz with a maximal elongation of 10% for 6 or 24 h by Flexcell FX-5000 Tension System (Flexcell Int. Corp.). Cardiomyocytes were divided into two groups to receive placebo or PIP3 micelles together with 500 nM VO-OHpic (PTEN inhibitor; #V8639, Millipore Sigma) and 30 μM PBP-10 (PIP2-binding peptide; #4611, Tocris Bioscience). After completion of the stretching protocol, cells were either frozen for protein isolation or fixed with paraformaldehyde, and later used to perform F-actin and G-actin double staining. The ratio between F-actin and G-actin staining intensities was represented as an index of actin polymerization. Protein isolated from frozen cells was utilized to assess the actin-depolymerizing activity.

### Statistical analysis

Sample sizes were calculated to be able to detect a moderate effect size (Cohen’s moderate; *α* = 5%, *β* = 10%, 90% power of the study) accounting for the expected death of animals (in survival surgeries). All data are shown as mean ± SEM. All statistical analyses were performed using SPSS software (Chicago, Illinois; Version 23). The effects of genotype and TAC were evaluated using one-way ANOVA followed by the Tukey’s post hoc test for multiple comparison testing. Unpaired Student’s *t* test (two-tailed) was used to compare two groups. Kaplan–Meier survival curves were analyzed using the log-rank (Mantel-Cox) test. Statistical significance is recognized at *p* < 0.05.

### Reporting summary

Further information on experimental design is available in the Nature Research Reporting Summary linked to this article.

## Supplementary information

Supplementary Information

Description of Additional Supplementary Files

Supplementary Data 1

Supplementary Movie 1

Supplementary Movie 2

Supplementary Movie 3

Supplementary Movie 4

Reporting Summary

## Data Availability

The data that support the findings of this study are available from the corresponding author upon reasonable request.
