# Supplementary material for: PI3Kα-regulated gelsolin activity is a critical determinant of cardiac cytoskeletal remodeling and heart disease
Source: Nat Commun. 2018 Dec 19;9:5390. doi: 10.1038/s41467-018-07812-8 (PMC6300608; doi:10.1038/s41467-018-07812-8)
Supplement: Supplementary file 2 — Description of Additional Supplementary Files [file 41467_2018_7812_MOESM2_ESM.docx]

**Description of Additional Supplementary Files**

**File Name:** Supplementary Movie 1

**Description:** Gelsolin N‐terminus bound with PIP2. Distances to amino acids of the

binding site (labeled in green) are given in angstroms in yellow.

**File Name:** Supplementary Movie 2

**Description:** Gelsolin C‐terminus bound with PIP2. Distances to amino acids of the

binding site (labeled in green) are given in angstroms in yellow.

**File Name:** Supplementary Movie 3

**Description:** Gelsolin N‐terminus bound with PIP3. Distances to amino acids of the

binding site (labeled in green) are given in angstroms in yellow.

**File Name: Supplementary Movie 4**

Gelsolin C‐terminus bound with PIP3. Distances to amino acids of the

binding site (labeled in green) are given in angstroms in yellow.

**File Name:** Supplementary Data 1

**Description:**Numerical Data for each Figure and Supplemental Figures.
