# Supplementary material for: PI3Kα-regulated gelsolin activity is a critical determinant of cardiac cytoskeletal remodeling and heart disease
Source: Nat Commun. 2018 Dec 19;9:5390. doi: 10.1038/s41467-018-07812-8 (PMC6300608; doi:10.1038/s41467-018-07812-8)
Supplement: Supplementary file 1 — Supplementary Information [file 41467_2018_7812_MOESM1_ESM.pdf]

PI3Kα regulated gelsolin activity is a critical determinant of cardiac cytoskeletal remodeling and heart disease

Patel and Zhabyeyev *et al.*

SUPPLEMENTARY INFORMATION

Supplementary Methods

Transverse aortic constriction. 8 – 8.5 weeks old male WT, PI3KαDN, PI3Kα$^{flox/flox}$, PI3Kα$^{flox/flox/Cre}$, *Gsn*$^{-/-}$ (GSNKO) and PI3KαDN/GSNKO mice weighing 20–25 g were used in these experiments as previously described[1-5]. Mice were anesthetized with isoflurane (#CP0406V2, Fresenius Kabi Canada). The skin was cleaned with Germex and Betadine. One dose of penicillin-streptomycin (10 mg kg^{-1}, 0.1 mL i.p.; #LS15140122, Fisher Scientific) was administered prior to start of surgery. Mice were placed supine and body temperature was maintained at 37°C with a heating pad. A horizontal skin incision of 1 cm in length was made at the level of second intercostal space, once the animal was in surgical plane of anesthesia (lack of reflex or response to toe-pinching). A 6-0 silk suture was passed under the aortic arch, a bent 27-gauge (27G) needle was then placed next to the aortic arch and the suture was snugly tied around the needle and aorta between the left carotid artery and the brachiocephalic trunk. The needle was quickly removed allowing the suture to constrict the aorta. The incision was closed in layers and the mice were allowed to recover on a warming pad until they were fully awake. Immediately after the surgery, mice received one dose of buprenorphine (#258-396-8, Millipore Sigma). Sham animals underwent the same procedure without the aortic banding. Measures were taken to avoid any infection to the mice.

Echocardiographic imaging and pressure-volume loop analysis. Transthoracic M-mode and Doppler echocardiographic examination at 2 weeks- and 9 weeks post-TAC were performed using the Vevo 770 high-resolution imaging system equipped with a 30-MHz transducer (#RMV-707B; VisualSonics, Toronto, Canada) as previously described[6-8]. Mice were placed on a heating pad and a nose cone with 0.75-1% isoflurane in 100% oxygen was applied. The temperature was maintained at 36.5 to 37.5°C. Ultrasound gel was placed on the chest of the anesthetized mouse. The ultrasound probe was placed in contact with the ultrasound gel and scanning was performed over 30 min. The temperature and heart rate (HR) were constantly monitored during the scanning. M-mode images were obtained for measurements of left ventricular (LV) wall thickness (LVWT), LVEDD, and LVESD. M-mode images were used to measure LV chamber size and wall thickness. LVEF, LVFS and VCFc were also calculated.

To measure LV pressure-volume relationship, 1.2F admittance catheter (Scisense Inc.) was used as previously described[9,10]. Briefly, mice were anesthetized with isoflurane, and an incision was made in the right carotid artery. A catheter was inserted into the incision and was advanced to the LV through the ascending aorta and aortic valve. The position of the catheter was monitored by pressure along with the magnitude and phase using ADvantage pressure-volume system (Scisense Inc.) and iworx data acquisition system (iWorx Systems Inc.) connected to the catheter. Once, the desired range for magnitude and phase was achieved, a baseline scan was performed to derive volume using Baan's equation, and the pressure-volume loop was obtained using the LabScribe2 software (iWorx Systems Inc., version 2.347000). The inferior vena cava was briefly occluded to obtain alterations in venous returns to derive end-diastolic pressure-volume relationships. The pressure-volume loops were analyzed using the LabScribe2 software.

Histological analyses, wheat germ agglutinin and F- and G-actin staining. Hearts were arrested in diastole with 1M KCl, fixed with 10% buffered formalin, and embedded in paraffin. Ten-micrometer thick sections were stained with picro-sirius red (PSR) or Masson trichrome to assess myocardial fibrosis and were visualized using fluorescence microscopy (Olympus IX81) and light microscopy (DM4000 B, Leica), respectively, as we have previously described[7,11]. Myocardial collagen contents were measured from the picrosirius red staining images using Metamorph Basic (Molecular Devices Inc., version 7.7.0.0) software.

Five-micrometer thick OCT embedded cryosections were stained with wheat germ agglutinin (WGA) to stain the cellular membrane and measure the myocyte cross-sectional area. Briefly, OCT embedded cryosections were fixed with 4% paraformaldehyde and washed in Dulbecco's Phosphate-Buffered Saline (DPBS; #A1285601, ThermoFisher). The sections were then permeabilized with 0.1% Triton X-100 (#9002-93-1, Millipore Sigma) in DPBS. After washing with DPBS for three times, the sections were stained with Oregon Green 488 conjugated WGA (#W6748, ThermoFisher) in 1.5% bovine serum albumin (BSA; #BAH66, Equitech Bio, Inc.) for 20 minutes at room temperature. The sections, after washing with DPBS for three times, were then mounted using Prolong Gold antifade mounting medium with DAPI (#D3571, ThermoFisher) and visualized under fluorescence microscope (Olympus IX81). Myocyte cross-sectional areas were measured by tracing areas congruent to the WGA stained myocyte membranes using Metamorph Basic (Version 7.7.0.0) software.

Five-micrometer thick OCT embedded cryosections were also stained with phalloidin and WGA to visualize cytoskeletal actin filaments (F-actin). Briefly, the cryosections were fixed in 4% paraformaldehyde, permeabilized with 0.1% Triton X-100 in DPBS and incubated with mixture of Alexa Fluor 488 conjugated phallodin (#A12379, ThermoFisher) and tetramethylrhodamine-WGA (#W849, ThermoFisher) in 1.5% BSA for 20 minutes at room temperature. The sections were then mounted using Prolong gold antifade mounting medium with DAPI to counterstain nuclei and visualized under the fluorescence microscope (Olympus IX81). The sections were washed three times with DPBS in between each step. Separately, the cryosections were double-stained with Alexa Fluor 488 conjugated phalloidin to stain F-actin and Alexa Fluor 594 conjugated DNAse I (#D12372, ThermoFisher) to stain the monomeric globular actin (G-actin) and visualized and imaged using fluorescence microscope (Olympus IX81). The images were morphometrically quantified using Metamorph Basic (Version 7.7.0.0) and F-actin to G-actin ratio was represented as an index of actin polymerization and compared between each group.

Immunofluorescence. IF staining was performed on 5 micrometer thick OCT embedded cryosections of LV. Briefly, cryosections were fixed with 4% formaldehyde for 20 minutes, and rehydrated with PBS for 30 minutes. LV sections were then permeabilized with 0.1% Triton-X100 in PBS for 5 minutes, followed by blocking with 4% bovine serum albumin for 1 hour. They were then incubated with primary antibody against p110α (#Sc-1331, Santacruz Biotechnologies; 1:100), N-cadherin (#610920, BD Biosciences; 1:100), β-catenin (#610154, BD Biosciences; 1:100), p-FAK (#MAB1144, Chemicon; 1:100), FAK (#Sc-558, Santacruz Biotechnologies; 1:50), gelsolin (#Sc-6406, Santacruz Biotechnologies; 1:100), alpha-smooth muscle actin (#Sc-53015, Santacruz Biotechnologies; 1:50), alpha-sarcomeric actin (#M0874, Dako; 1:50), and PIP3 (#Z-P345, Echelon Biosciences; 1:25; 20 μg mL^{-1} final concentration) overnight at 4°C in a humidified chamber. Sections were incubated with different fluorophore conjugated secondary antibodies (Invitrogen, USA) at 37°C for 1 hour in a humidified chamber. LV sections were visualized under fluorescence microscope (Olympus IX81) after mounting with ProlongGold antifade mounting medium with DAPI (#P36931, ThermoFisher). The sections were washed with PBS, three times for 5 minutes, in between the steps.

Protein extraction and immunoblotting. Total protein extraction and immunoblotting (IB) were performed as previously described[2,5-7]. Typically, 100 μg of total protein was used for IB of phospho- (Thr308; #9275, Cell Signaling Technology) and total Akt (#9272, Cell Signaling Technology), phospho- (Ser16/Thr17; #A010-12, Badrilla) and total Phospholamban (#A010-14, Badrilla), SERCA2a (#sc-53010 Santa Cruz), and calpain (#2556s, Cell Signaling Technology).

Immunoprecipitation and immunoblot analysis of Gelsolin and p110α. Human and murine heart tissues (400 mg) were lysed in 400 μl of CelLytic™ M lysis buffer (#C2978, Millipore Sigma) containing 1x cOmplete Protease (#11697498001, Sigma-Aldrich) and PhosSTOP Phosphatase

(#4906845001, Millipore Sigma) inhibitor cocktails. Protein concentration of the whole tissue lysates was measured using Bradford protein assay kit I (#5000001, Bio-Rad Canada). A total of 50 μl of the tissue lysates was saved. The same amount of total protein was applied to DynaMag™ Trial Magnet beads (ThermoFisher) pre-incubated with a Goat polyclonal anti-Gelsolin antibody (#sc-6406, Santa Cruz) or a Rabbit monoclonal anti-P110α antibody (#MA5-14870, ThermoFisher) to immunoprecipitate Gelsolin and P110α, respectively. Immunoprecipitated samples were washed three times with PBS buffer containing 0.01% Triton X-100. The immunoprecipitated proteins were then eluted from the beads by addition of 4× SDS-PAGE sample buffer (#1610747, Bio-Rad Canada) containing 10% β-mercaptoethanol. The eluted samples and the same amount of the whole tissue lysates were subjected to SDS-PAGE and transferred to immobilon@P PVDF membranes (#IPVH00010, Millipore Sigma) by electroblotting. Immunoblotting was performed using rabbit anti-Gelsolin polyclonal antibody (#sc-48769, Santa Cruz), and goat anti-P110α polyclonal antibody (#sc-1331, Santa Cruz) to detect immunoprecipitated Gelsolin and P110α, respectively. Antibody binding was detected using anti-goat or anti-rabbit IgG (HRP).

TaqMan Real-time PCR. RNA expression levels of various genes were determined by TaqMan Real-time PCR as previously described[1,5,6]. Total RNA was extracted from flash-frozen tissue using TRIzol extraction protocol, and cDNA was synthesized from 1 μg RNA by using random hexamers. For each gene, a standard curve was generated using known concentrations of cDNA (0.625, 1.25, 2.5, 5, 10 and 20 μg) as a function of cycle threshold (CT). Expression analysis of the reported genes was performed by TaqMan Real-time PCR using ABI 7700 Sequence Detection System (Conquer Scientific). The SDS2.2 software (integral to ABI7700 real-time machine) fits the CT values for the experimental samples and generates values for cDNA levels. All samples were run in triplicates in 384 well plates. 18S rRNA was used as an endogenous control.

Isolated cardiomyocyte contractility. For measurement of contractility, cardiomyocytes were isolated as described previously[5]. After isolation myocytes were kept in perfusion buffer solution (pH 7.4). An aliquot of isolated cardiac myocytes were transferred in a glass-bottomed recording chamber on top of inverted microscope (Olympus IX71) and allowed to settle for 5-6 min. Cells were superfused at a rate of 1.5-2 mL min^{-1} with modified Tyrode's solution (containing in mmol L^{-1}: 135 NaCl, 5.4 KCl, 1.2 $CaCl_2$, 1 $MgCl_2$, 1 NaH_2PO_4, 10 Taurine, 10 HEPES, 10 glucose; pH 7.4 with NaOH; all purchased from Millipore Sigma). The perfusion solutions were heated to in-bath temperature of 35-36°C using in-line heater (SH-27B, Harvard Apparatus) controlled by automatic temperature controller (TC-324B, Harvard Apparatus). Quiescent rod-shaped myocytes with clear striations were selected for study. Platinum-wire electrodes were placed near the cell just outside of the microscope view at 400X magnification. Myocytes were paced at 1 Hz with voltage of 3-4 V (50% above threshold) and pulse duration of 2.5 ms using S48 stimulator (Grass Technology). Sarcomere length was estimated in real time from images captured at a rate 200 frame s^{-1} via 40X objective (UAPO 40X3/340, Olympus) using high-speed camera (IMPERX IPX-VGA-210, Aurora Scientific). Sarcomere length was calculated by HVSL software v. 1.75 (Aurora Scientific) using auto-correlation function (ACF/Sine-fit) algorithm and stored in a file for analysis at a later time. Myocytes were paced for at least 2 min. Only recordings of myocytes that produced stable contractions of similar amplitude and kinetics at a steady-state (past 2 min) were selected for analysis. At about 2 min of stimulation time, 10 consecutive contractions were selected and averaged to reduce noise and make calculations of derivatives more precise. Averaged contraction was used to calculate fractional shortening (FS), relaxation times (t50 and t90), and ±dL dt^{-1}. Calculations were performed in Origin 8.5 (OriginLab) using custom-made script of built-in LabTalk language.

Measurement of L-type Ca^{2+} current ($I_{Ca,L}$). Aliquots of solution containing murine cardiomyocytes were placed in a bath mounted on top of an inverted microscope (Olympus IX71, Olympus, Canada), and rod-shaped quiescent myocytes were selected for study. The myocytes were superfused with K^+-free modified Tyrode's solution (containing in mmol L^{-1}: NaCl, 135; $CaCl_2$, 1.2; $MgCl_2$, 1; NaH_2PO_4, 1; HEPES, 10; taurine, 10; glucose, 10; all purchased from Millipore Sigma) at 35-36°C. Pipettes with resistance of 1.5-2.5 MOhm were filled with Cs^+ pipette solution (containing in mmol L^{-1}: CsOH, 110; CsCl, 30; L-Aspartic acid, 110; MgATP, 5; EGTA, 5; HEPES, 10; all purchased from Millipore Sigma). Currents were recorded from whole cell in the ruptured patch configuration using Multiclamp 700B amplifier (Molecular Devices, USA) in voltage-clamp mode. Signal was digitized by 16-bit analog-digital board DigiData 1440A (Molecular Devices, USA) under control of pClamp 10 software (Molecular Devices, USA) and stored for offline analysis. Ca^{2+} current ($I_{Ca,L}$) was elicited by step depolarizations from holding potential of –40 mV to the test potential 0 mV for 200 ms. Peak $I_{Ca,L}$ was defined as the difference between peak inward current and the steady state current at the end of the test pulse.

***In Silico* Modeling of gelsolin and its individual N- and C-terminus domain.** Human gelsolin has a solved X-ray crystal structure (PDB ID: 3FFN)[12], but several residues (1-49, 260-264, 756-782) were missing in the crystal structure. Comparative homology modeling was used to construct the full length structure of human gelsolin using this crystal structure (PDB ID: 3FFN) as a template. The crystal structure of calcium activated human gelsolin N-terminus complexed with actin (PDB ID: 3FFK)[12] also contains missing residues. We have used this structure as a reference to build the full length model of calcium activated human gelsolin N-terminus bound with actin. Human gelsolin C-terminus has its both calcium bound (PDB ID: 1P8X)[13] and actin bound structure (PDB ID: 1H1V) [14] with several missing residues. So, we have built full length model for all of them. Models were built using Modeller 9.14 (http://salilab.org/modeller/)[15,16].

Ligand 3D structure preparation. 2D structure of both the Phosphatidylinositol 4, 5-bisphosphate (PIP2) and phosphatidylinositol (3, 4, 5)-trisphosphate (PIP3) have been drawn using ChemDraw software. 3D structures of those were calculated using ChemUltra3D[17].

Docking of PIP2 and PIP3 with N- and C-terminus domain. To model the gelsolin N-terminus-PIP2 complexes, we used the knowledge-based Autodock Vina docking method[18]. The grid for docking was developed using structurally aligned Horse N-terminus-ATP bound structure (PDB ID: 2FGH)[19] with human gelsolin N-terminus. Using the built-in Grid map option, we have prepared the axes dimensions and center points for performing the docking of PIP2 with Human gelsolin N-terminus. For modeling the human gelsolin C-terminus bound with PIP3 we have used the active site grid dimension of N-terminus bound PIP2. Structural alignment between the N-terminus and C-terminus domains of human gelsolin revealed that the two domains are homologous to each other. The N-terminus G1-G3 sub domains resembles structurally with the G4-G6 of C-terminus. Keeping this knowledge in mind, we have used the binding grid of PIP2 with N- and C-terminus as same as for PIP3. In each docking experiment, we have used 10 conformations of the ligand. After selecting the conformations of the ligand that best fit with human gelsolin N-terminus and C-terminus domain. We have refined the complex based on the active site residues using COOT[20]. We optimized the substrate residues by using rotamer selection and regularize zone tools.

MD simulation. All molecular dynamics (MD) simulations of the PIP2 and PIP3 bound gelsolin complexes were performed using GROMACS 5.1.2 software package[21], utilizing the GROMOS96 43a1 force field. The starting structures for the 50 ns simulations of the PIP2 and PIP3 each complexed with both the $GSN_{N\text{-}ter}$ and $GSN_{C\text{-}ter}$ were obtained from the molecular docking of PIP2 and PIP3 to the catalytic domain of gelsolin, respectively as described above. Dundee PRODRG

Server [22] was used for generating the topology and parameter files of PIP2 and PIP3 molecules for MD simulation. Each protein-ligand complex was placed in the center of a cubic box with specific dimensions containing definite simple point charge water molecules for the individual complex as shown Supplementary Table 3. Some water molecules were replaced by ions to neutralize the systems.

All starting structures were subsequently energy minimized with a steepest descent method for 5000 steps. The results of these minimizations produced the initial structures for the position restraining MD simulations. These structures were subjected to 200 ps of position restrained simulations, where the protein and the ligands were kept fixed and water molecules were allowed to settle down around the ligand protein complex. These structures were finally used for the 50 ns of MD simulations in the aqueous solution at 300K temperature. The simulations were performed under two successive equilibration phases. At the first phase of equilibration, constant number of particles (N), volume (V) and temperature (T) i.e. NVT ensemble is maintained for all the complex systems. In the second phase, a constant number of particles (N), pressure (P) and temperature (T) i.e. NPT ensemble were maintained. The SETTLE algorithm[23] was used to constrain the bond length and angle of the water molecules, while the LINCS algorithm[24] was used to constrain the bond length of the peptides. The long range electrostatic interactions were calculated by the Particle Mesh Ewald (PME) method[25] with interpolation order of 4 and a grid spacing of 12 Å. A constant pressure of 1 bar was applied with a coupling constant of 1.0 ps; peptide, ligand, water molecules and ions were coupled separately to a bath at 300 K with a coupling constant of 0.1 ps. The periodic boundary conditions were applied and the equation of motion was integrated at time-steps of 2 fs. All the systems were treated in position restrained environment. The SHAKE algorithm was used to restrain bond lengths involving hydrogen atoms[26]. The van der Waals interactions were treated by using a cutoff of 12 Å. Finally, 50 ns production MD run were performed under the normal temperature and pressure with the coupling time constants of 0.1 ps and 1.0 ps respectively. All the simulations were performed with a time step of 2 fs, and the coordinates were saved every 1 ps. The analyses were carried out using the programs within the GROMACS 5.1.2 package, and the figures were drawn in PyMol[27], VMD[28], Chimera[29] and Xmgrace[30].

Normal mode analysis. Normal mode analysis (NMA) is one of the techniques used to determine large-scale motions in protein. By this approach we can detect significant conformational change of protein dynamism during ligand bound and unbound state. In NMA, mainly the backbone motion of the protein is considered to observe any loop movement or local domain motions in protein. This method includes a unique collection of multiple elastic network model (ENM) force-fields and automated ensemble analysis[31,32]. Using NMA we can also track the most probable transition pathway and hence different intermediate conformations between two significantly distinguished states of protein. NMA also facilitates Root mean square inner product (RMSIP) as an overlap measure between conformational spaces explored during the simulations[33]. This tool presents all possible conformationally distinguished modes of a protein by calculating the deformation energy between successive modes which help in understanding the role of each residue in the transition from one conformer to another.

Principal component analysis (PCA). We have analyzed protein-ligand dynamism of all the complex systems of gelsolin bound with PIP2 and PIP3 in aspects of principal component analysis (PCA)[34-36]. PCA is a standard tool in statistical mechanics, which allows us to determine the correlated motions of the residues to a set of linearly uncorrelated variables called principal components. This method is based on the construction of the covariance matrix of the coordinate fluctuations of the simulated proteins. The covariance matrix is diagonalized to obtain the eigenvectors and eigenvalues that provide information about correlated motions throughout the protein. We have compared the eigenvalues for first two eigenvectors of principal component

(PC1 and PC2) of mutant residues in their corresponding systems. Average structure as a function of each principal component has been investigated to determine the role of specific mutation on protein-ligand binding mode and interaction affinity.

Isolation, culture and stretching of adult cardiomyocytes. Adult murine left LV cardiomyocytes were isolated and cultured as described previously[11,37]. Cardiomyocytes were cyclically stretched at 1 Hz and a maximal elongation of 10% for 6 or 24 hours with Flexcell FX-5000 Tension System (Flexcell Int. Corp.). After the completion of stretching protocol the cells were imaged under light microscope (DM4000 B, Leica) to assess the cell viability. Media were collected to perform the LDH assay to assess the cell death. The cells were then washed with PBS and fixed with 2% paraformaldehyde for 20 minutes followed by 3 washings with PBS. The fixed cardiomyocytes were then used to perform the F-actin and G-actin double staining. The ratio between F-actin and G-actin staining intensities was represented as an index of actin polymerization.

	WT – Sham	WT – 9 Wk TAC	WT – 18 Wk TAC	GSNKO – Sham	GSNKO – 9 Wk TAC	GSNKO – 18 Wk TAC
n (Biological replicates)	8	9	8	8	10	10
HR (bpm)	413±33	437±25	457±18	426±25	452±11	431±22
EDP (mmHg)	8.7±2.33	24.5±0.5*	25.09±4.42*	8.8±0.9	9.89±0.1#	15.5±2.18*,#
ESP (mmHg)	80.2±7.7	134.3±1.75*	137.6±8.9*	95.4±2.6	127.01±13*	134.43±1.9*
EDV(µl)	26.24±2.15	51.83±4.89*	62.7±13.8*	29.9±0.9	35.34±3.42#	34.44±3.76#
ESV (µl)	3.92±0.29	25.58±2.89*	43.19±12*	9.40±0.7	10.87±2.14#	11.253±2.54#
SV(µl)	22.31±2.01	26.24±3.58	19.69±2.79	20.59±0.14	24.47±3.37	23.19±2.03
CO (ml min^{-1})	9023±542	10811±358*	8754±1115	8832±501	10992±1505*	10196±1274*,#
EF (%)	84.8±1.2	50.56±3.89*	35.63±4.7*	68.62±1.64	68.73±5.46#	69.31±4.31#
dP dt_{max}^{-1} (mmHg s^{-1})	6058±1091	6896±689	6831±364	7309±333	6746±564	6942±225
SW (m Joules)	0.2388±0.03	0.483±0.04*	0.3661±0.06*	0.2655±0.01	0.406±0.04*	0.4251±0.03*
dP/dt_{max}/EDV (mmHg s^{-1} $µl^{-1}$)	235.32±47.93	133.06±15.69*	140.41±21.78*	244.25±18.46	200.12±25.53#	220.42±26.23#
ESPVR (mmHg $µl^{-1}$)	6.6±0.7	2.843±0.3*	2.835±0.7*	6.73±0.05	7.28±0.53#	5.06±1.38#
τ (Glantz) (ms)	11.625±1.21	13.861±2.81	17.52±2.12*	9.98±0.23	11.82±1.09	10.69±0.89#
EDPVR (mmHg $µl^{-1}$)	0.106±0.012	0.061±0.002*	0.041±0.012*	0.111±0.014	0.096±0.018#	0.092±0.018#

Supplementary Table 1. Pressure-Volume loop assessment of cardiac function in WT and GSNKO hearts in response to sham or chronic pressure-overload. HR, heart rate; EDP, end diastolic pressure; ESP, end systolic pressure; EDV, end diastolic volume; ESV, end systolic volume; SV, stroke volume; CO, cardiac output; EF, left-ventricular ejection fraction; dP/dt_{max}/EDV, maximal rate of change in left-ventricular pressure adjusted by EDV; ESPVR, end systolic pressure volume relationship; τ (Glantz), Glantz relaxation constant; EDPVR, end-diastolic pressure volume relationship. *P<0.05 compared with the respective sham group, #P<0.05 compared with corresponding WT – 9 Wk or 18 Wk TAC group as determined by one-way ANOVA analysis.

N-terminus	4,5 PIP2	Distance*	N-terminus	3,4,5 PIP3	Distance*
Lys166NZ	OP3	3.9	Lys135NZ	O11	3.4
Arg168NH1	O5	3.1	Lys166NZ	OP3	3.4
Arg168NH1	O52	2.9	Arg168NH1	OP3	3.9
Arg168NH1	O53	3.1	Arg168NH2	OP3	3.5
Arg169NH1	OP3	3.9	Arg169NH1	O5	3.0
Arg169NH2	OP3	3.4	Arg169NH1	O52	2.9
Arg172N	O7	2.7	Arg169NH1	O53	3.0
Glu263OE2	OP2	3.4	Arg172NH1	O33	3.8
			Arg172NH2	O33	4.0
			Arg172N	O7	3.1
			Glu263OE1	OP2	3.4
			Glu263OE2	OP2	3.0
			Gln349NE2	O43	4.0
**C-terminus**	**4,5 PIP2**	**Distance***	**C-terminus**	**3,4,5 PIP3**	**Distance***
Lys530NZ	O42	3.5	Lys530NZ	O42	3.4
Arg569NH2	O41	2.8	Arg569NH2	O41	2.7
Arg569NH2	O42	3.3	Arg569NH2	O42	3.2
Arg652NH2	O2	3.4	Arg652NH2	O2	3.4
Ser654OG	OP3	2.9	Ser654OG	OP3	2.9
Pro655O	OP2	2.6	Pro655O	OP2	2.6
Pro655O	OP3	3.1	Pro655O	OP3	3.2
Lys660O	O7	2.8	Arg656NH1	O3	2.8
His665 NE2	O11	2.9	Arg656NH1	O32	2.7
Asp705OD1	O51	2.8	Arg656NH1	O41	2.7
Asp705OD1	O52	2.8	Leu657N	O33	2.7
Asp705OD2	O43	2.9	Lys658NZ	O31	3.2
			Lys658NZ	O32	2.6
			Lys660NZ	OP2	2.9
			Lys660O	O7	2.8
			Lys661NZ	O31	2.6
			Lys661NZ	O4	3.5
			Lys661NZ	O43	3.5
			His665NE2	O11	3.0
			Asp705OD1	O52	2.8
			Asp705OD2	O43	3.1
			Arg738NH1	O52	2.8
			Arg738NH1	O53	3.4
			Arg738NH2	O52	2.6

Supplementary Table 2. Comparative molecular interactions of PIP2 and PIP3 with N- and C-terminus gelsolin. * All the Distances are in Angstrom (Å).

System	Box size (nm)	Number of Solvent Mols.	Ions added
**$GSN_{N\text{-}ter}$ + PIP2**	9.66*9.66*9.66	28118	1 Cl^-
**$GSN_{N\text{-}ter}$ + PIP3**	9.66*9.66*9.66	28108	1 Cl^-
**$GSN_{C\text{-}ter}$ + PIP2**	8.91*8.91*8.91	21740	7 Na^+
**$GSN_{C\text{-}ter}$ + PIP3**	8.91*8.91*8.91	21748	7 Na^+

Supplementary Table 3. MD simulation parameters used for the complex of N-ter and C-ter gelsolin bound with PIP2 and PIP3.

Genes	Primers & Probes	Sequence
ANF	Forward Primer:<br>Reverse Primer:<br>Probe:	5'-GGA GGA GAA GAT GCC GGT AGA-3'<br>5'-GCT TCC TCA GTC TGC TCA CTC A-3'<br>5'-FAM-TGA GGT CAT GCC CCC GCA GG-TAMRA-3'
BNP	Forward Primer:<br>Reverse Primer:<br>Probe:	5'- CTG CTG GAG CTG ATA AGA GA-3'<br>5'- TGC CCA AAG CAG CTT GAG AT-3'<br>5'- FAM-CTC AAG GCA GCA CCC TCC GGG-TAMRA-3'
ß-MHC	Forward Primer:<br>Reverse Primer:<br>Probe:	5'-GTGCCAAGGGCCTGAATGAG-3'<br>5'-GCAAAGGCTCCAGGTCTGA-3'<br>5'-ATCTTGTGCTACCCAGCTCTAA-3'
α-skeletal muscle actin	Forward Primer:<br>Reverse Primer:<br>Probe:	5'-CAGCCGGCGCCTGTT-3'<br>5'-CCACAGGGCTTTGTTTGAAAA-3'<br>5'-FAM-TTGACGTGTACATAGATTGACTCGTTTTACCTCATTTTG- TAMRA-3'
TNFα	Forward Primer:<br>Reverse Primer:<br>Probe:	5'- ACAAGGCTGCCCCGACTAC-3'<br>5'- TTTCTCCTGGTATGAGATAGCAAATC-3'<br>5'-FAM-TGCTCCTCACCCACACCGTCAGC-TAMRA-3'
TGFß1	Forward Primer:<br>Reverse Primer:<br>Probe:	5'- CCTGCAAGACCATCGACATG-3'<br>5'- ACAGGATCTGGCCACGGAT-3'<br>5'- FAM-CTGGTGAAACGGAAGCGCATCGAA-TAMRA-3'
Pro-col I-α1	Forward Primer:<br>Reverse Primer:<br>Probe:	5'-CTTCACCTACAGCACCCTTGTG-3'<br>5'-TGACTGTCTTGCCCCAAGTTC-3'<br>5'-FAM-CTGCACGAGTCACACC-TAMRA-3'
Pro-col III-α1	Forward Primer:<br>Reverse Primer:<br>Probe:	5'- TGTCCTTTGCGATGACATAATCTG-3'<br>5'- AATGGGATCTCTGGGTTGGG-3'<br>5'-FAM- ATGAGGAGCCACTAGACT-TAMRA-3'
18s		TaqMan™ Ribosomal RNA Control Reagents (#4308329, Thermofisher Scientific)

Supplementary Table 4. List of TaqMan primers and probes.

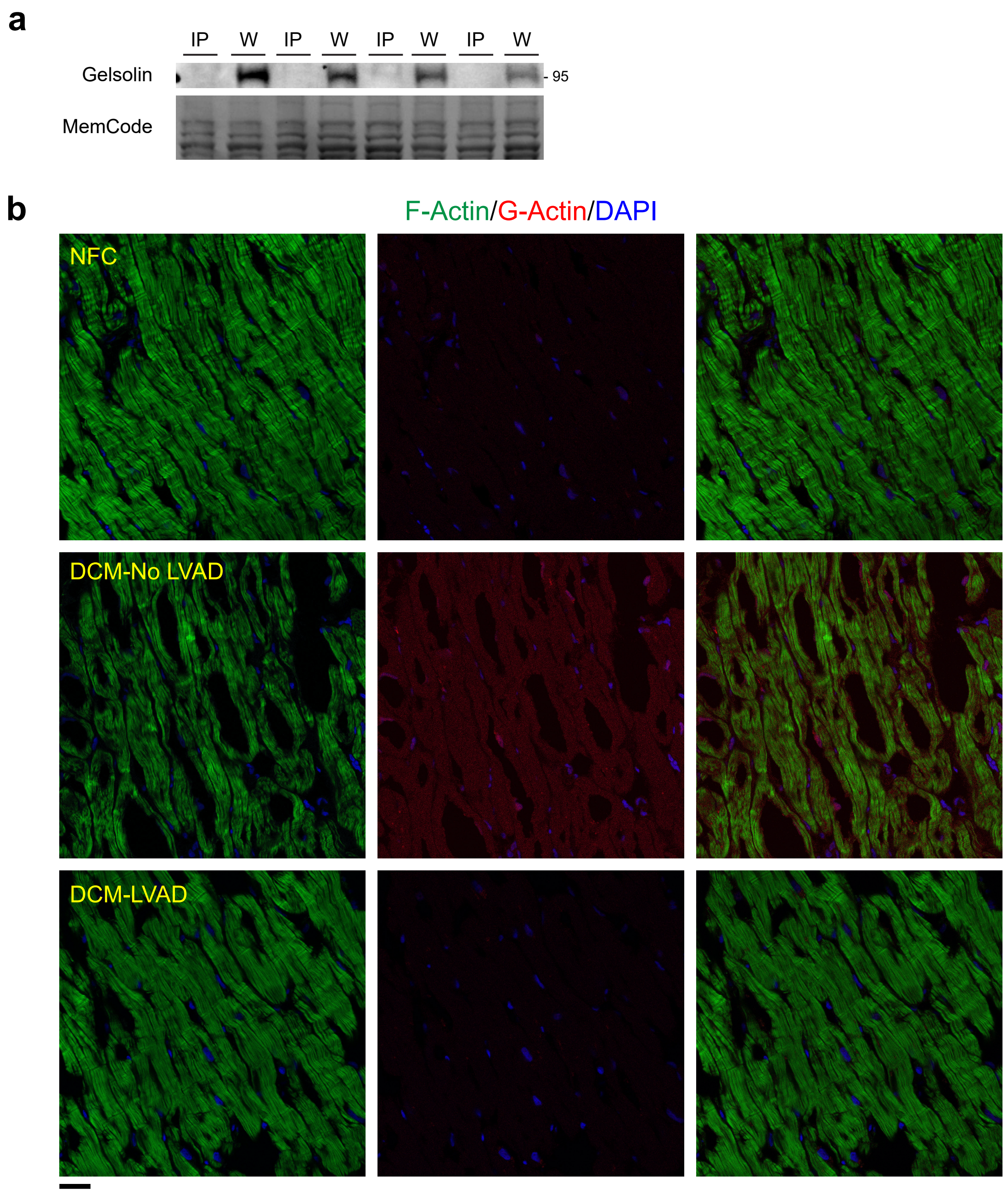

Supplementary Figure 1. Role of gelsolin in human dilated cardiomyopathy and heart failure. **a** Gelsolin immunoprecipitation using human dilated cardiomyopathy heart samples. **b** Reverse cytoskeletal remodeling in response to LVAD placement. Representative F-actin and G-actin staining images showing increased actin depolymerization in the explanted human hearts with dilated cardiomyopathy which was reversed by the placement of LVAD. Data represent means ± s.e.m. Biological replicates: n=4. Scale bar shows 25 μm (b).

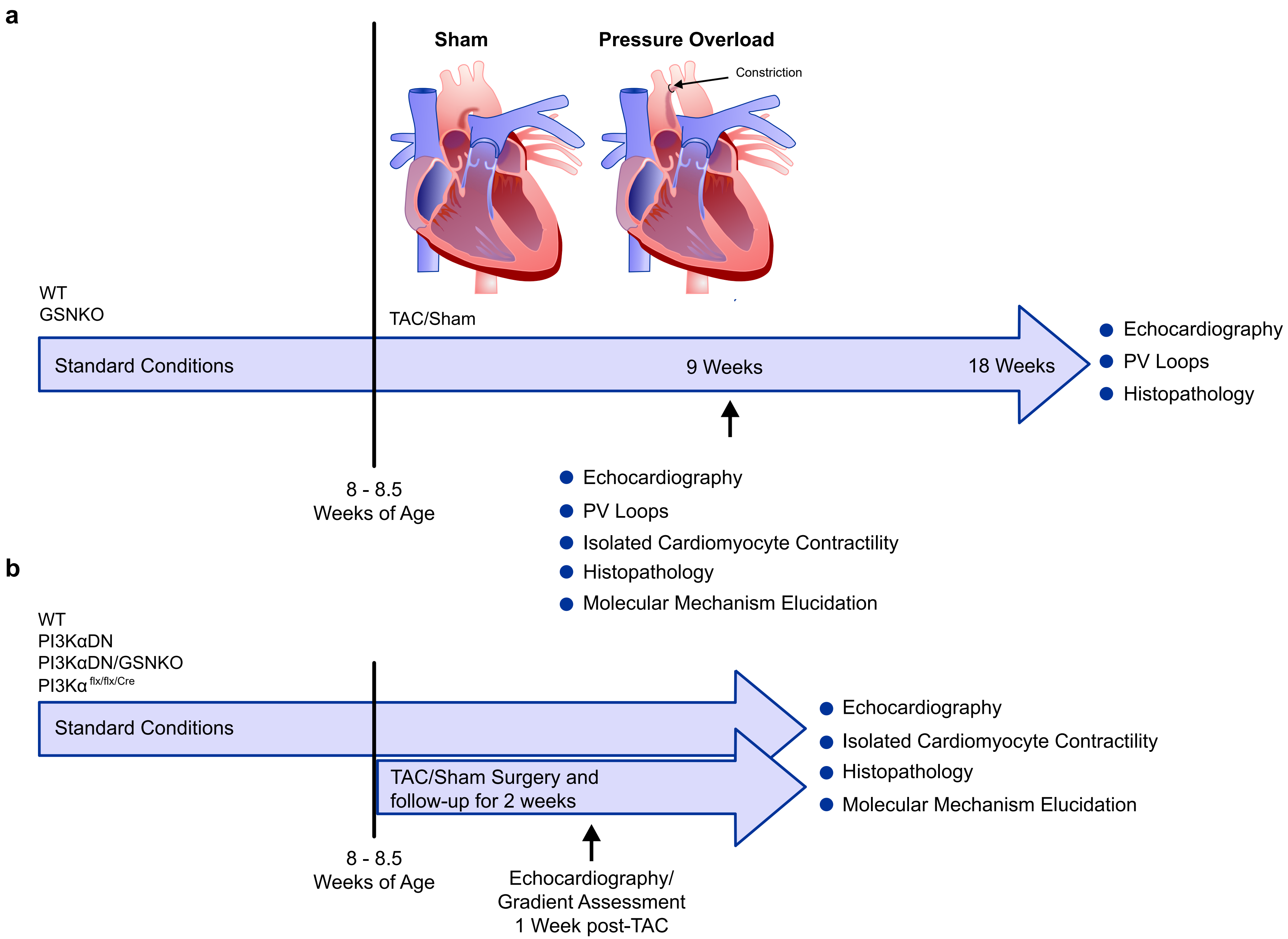

Supplementary Figure 2. Schematic of experimental plan. **a** WT and GSNKO mice were subjected to the transverse aortic constriction or sham surgery. Functional analyses were carried out at the end of 9 or 18 weeks of surgery and the mechanistic experiments were performed at 9 weeks after surgery. **b** WT, PI3KαDN, PI3Kα$^{flx/flx}$ Cre, and PI3KαDN/GSNKO mice were subjected to the transverse aortic constriction or sham surgery. Their transverse aortic gradient was evaluated at 1 week after surgery and the functional and mechanistic experiments were performed at 2 weeks after surgery.

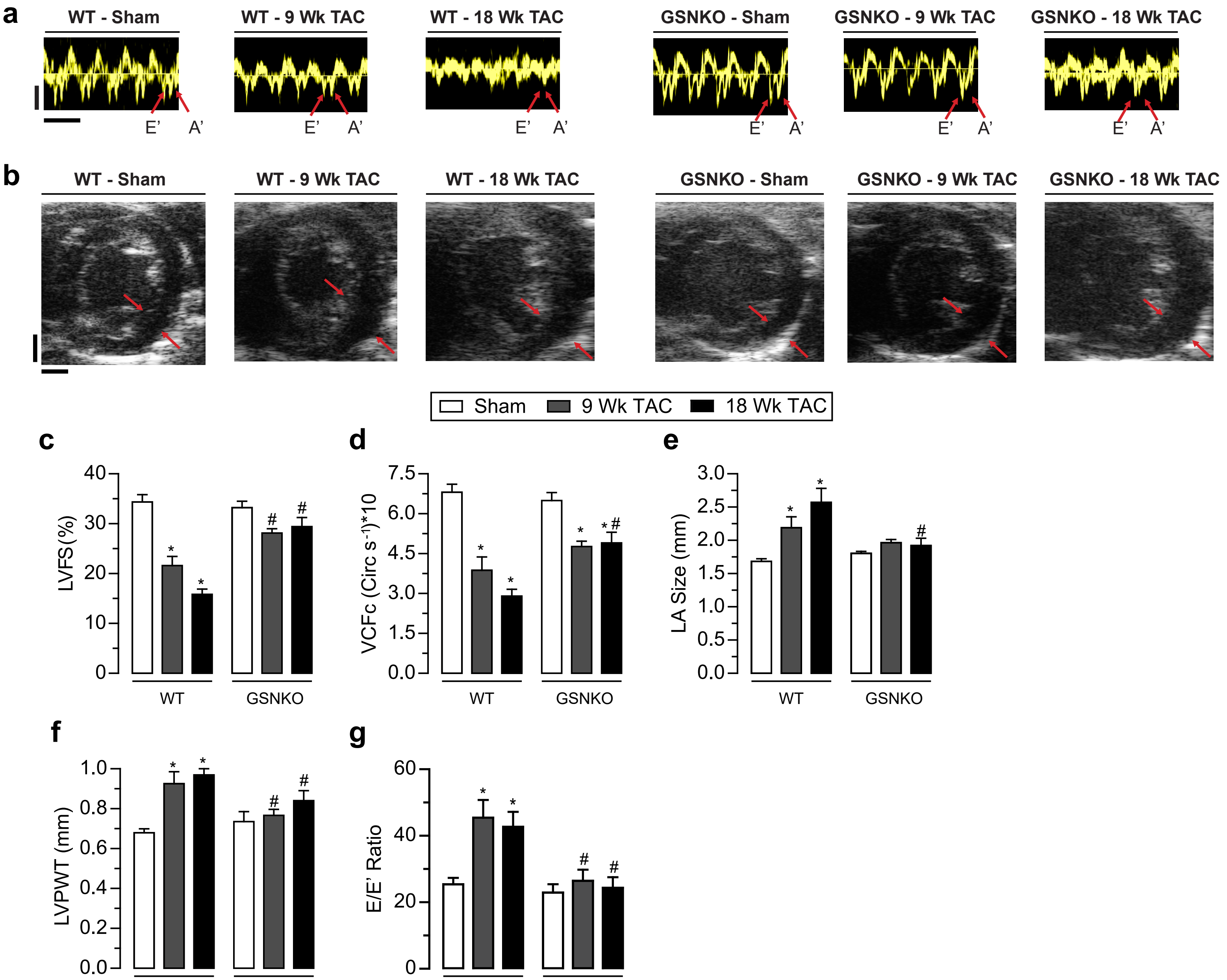

Supplementary Figure 3. Loss of gelsolin alleviates pressure overload-induced cardiac dysfunction. **a-b** Echocardiographic assessment using tissue Doppler imaging (a) and B-mode images of left ventricle (b). **c-f** Quantitative evaluation of cardiac function showing substantially decreased LV fractional shortening (LVFS; c) and velocity of circumferential fiber shortening (VCFc; d) along with increased LA size (e), LV posterior wall thickness (LVPWT; f) and E/E' ratio, a hallmark of diastolic function (g) in WT mice in response to 9 and 18 weeks of pressure overload. Loss of gelsolin attenuated the pressure overload-induced heart failure. Data represent means ± s.e.m. *P<0.05 compared with the respective control groups (Sham), #P<0.05 compared with respective WT – 9 Wk or 18 Wk TAC group as determined by one-way ANOVA analysis (c-g). Biological replicates: n=12. Scale bars show 2 cm s^{-1} (y-axis of a), 150 ms (x-axis of a), and 1 mm (x and y axes of b).

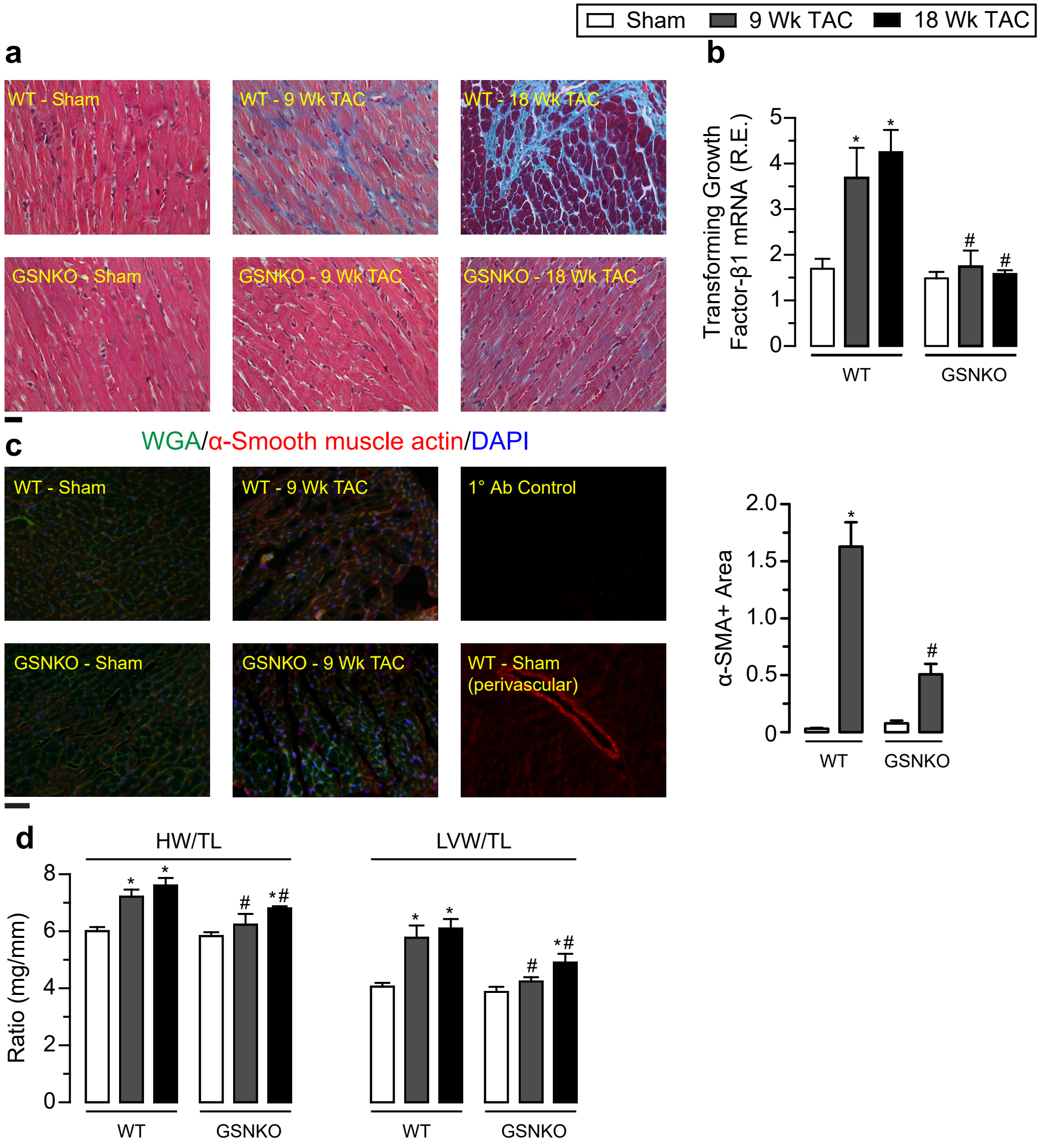

Supplementary Figure 4. Loss of gelsolin mitigates fibrosis in pressure overload-induced HF with reduced ejection fraction. **a-b** Masson trichrome staining images (a) and mRNA expression of TGF-β1 (b) showing increased cardiac fibrosis in WT hearts in response to pressure overload. Loss of gelsolin attenuated the pressure overload-induced cardiac fibrosis. **c** Representative immunofluorescence images and quantification of the alpha-smooth muscle actin-positive area (c) showing increased alpha-smooth muscle actin staining in WT hearts in response to 9 weeks pressure overload, whereas loss of gelsolin attenuated pressure overload-induced alpha-smooth muscle actin expression. Primary antibody control and perivascular region staining were used as negative and positive controls, respectively, for immunofluorescence staining of the alpha-smooth muscle actin. **d** Heart weight and left ventricle (LV) weight to tibial length ratio showing the attenuation of 9 and 18 weeks of pressure overload-induced cardiac hypertrophy with the loss of gelsolin. α-SMA: alpha-smooth muscle actin. Data represent means ± s.e.m. *$P<0.05$ compared with the respective control groups (Sham), #$P<0.05$ compared with respective WT – 9 Wk or 18 Wk TAC group as determined by one-way ANOVA analysis (b-d). Biological replicates: n=4 (a, c), n=10 (b) and n=12 (d). Scale bar shows 50 µm (a, c).

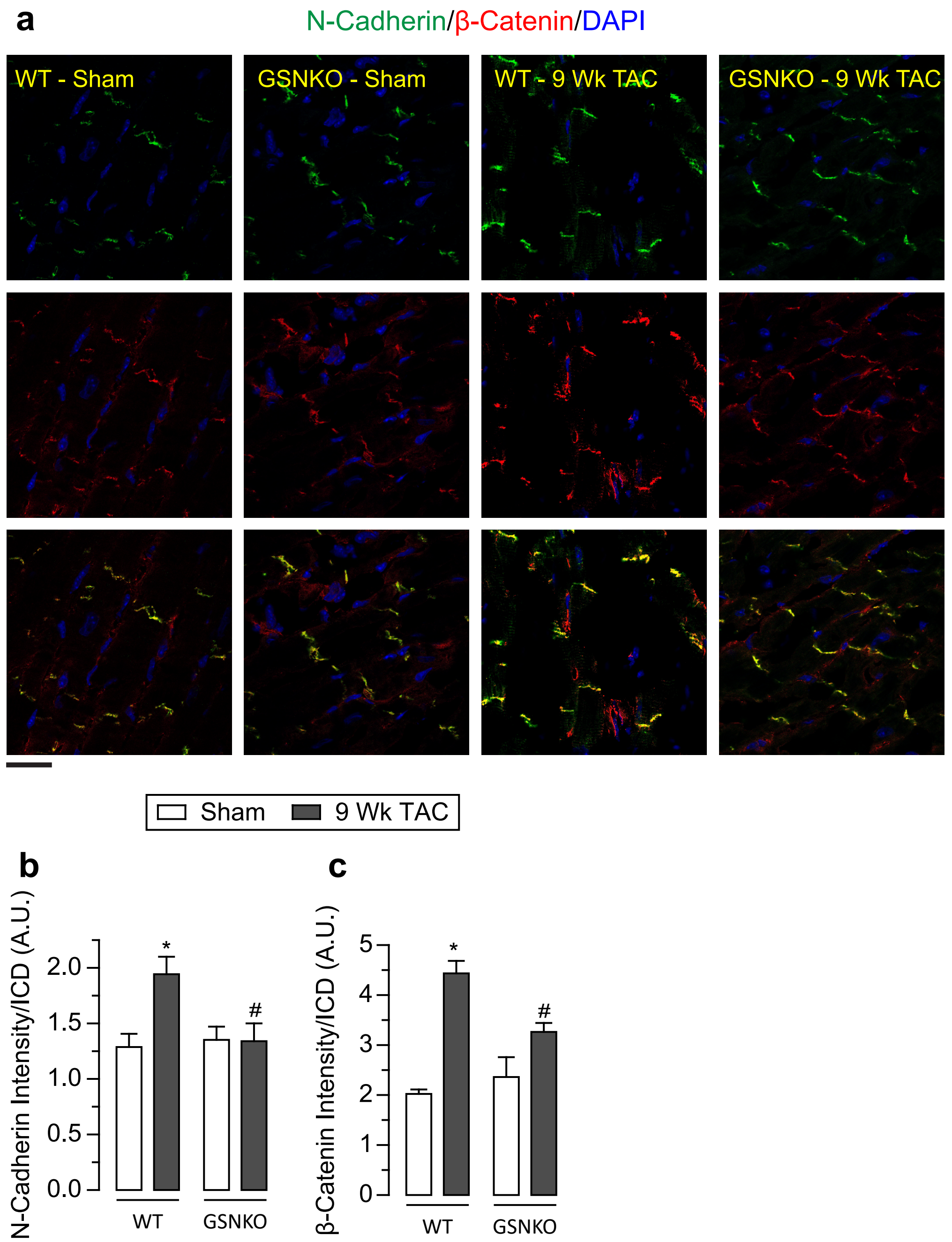

Supplementary Figure 5. Attenuation of pressure overload-induced upregulation of cardiac mechano-sensor proteins with loss of gelsolin. **a-c** Representative immunofluorescence images showing upregulation of myocardial levels of N-Cadherin (a-b) and β-catenin (a, c), mechano-sensors at intercellular junctions, in the WT hearts in response to pressure overload-induced biomechanical stress. Loss of gelsolin attenuated the pressure overload-induced upregulation of cardiac mechano-sensor proteins. Data represent means ± s.e.m. *P<0.05 compared with the respective control groups (Sham), #P<0.05 compared with respective WT – 9 Wk TAC group as determined by one-way ANOVA analysis (b-c). Biological replicates: n=4. Scale bar shows 25 µm (a).

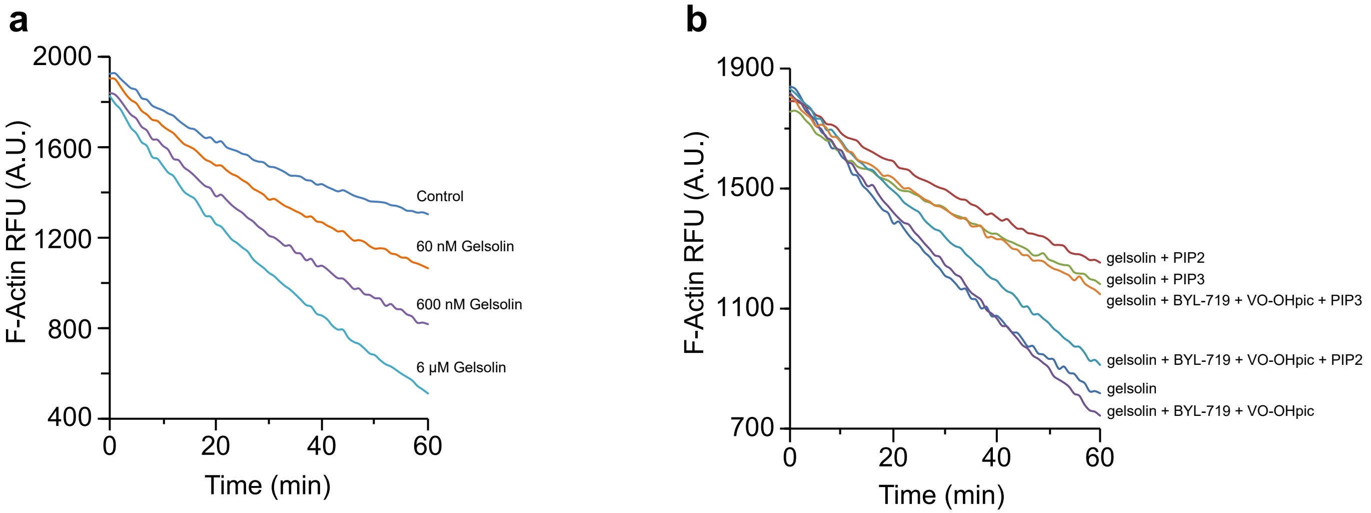

Supplementary Figure 6. Actin severing activity assay showing the decreasing relative fluorescence unit for pyrene-labelled actin in response to actin depolymerization. **a** Time course of the fluorescence showing the concentration-dependent effect of recombinant porcine cytosolic gelsolin, spiked in gelsolin-null heart tissue homogenate, on actin depolymerization assay. **b** Actin depolymerization assay showing inhibition of actin severing activity of recombinant porcine cytosolic gelsolin, in gelsolin-null heart tissue homogenate, by phosphatidylinositol-3,4,5-triphosphate (PIP3) in the presence of PTEN inhibition using VO-OHpic. Activity assay showed that phosphatidylinositol-4,5-triphosphate (PIP2) mediated inhibition of gelsolin actin severing activity assay is blocked by BYL-719 and VO-OHpic, suggesting that the conversion to PIP3 is necessary for the PIP2-mediated inhibition of gelsolin actin severing activity. Biological replicates: n=6 (a) and n=9 (b).

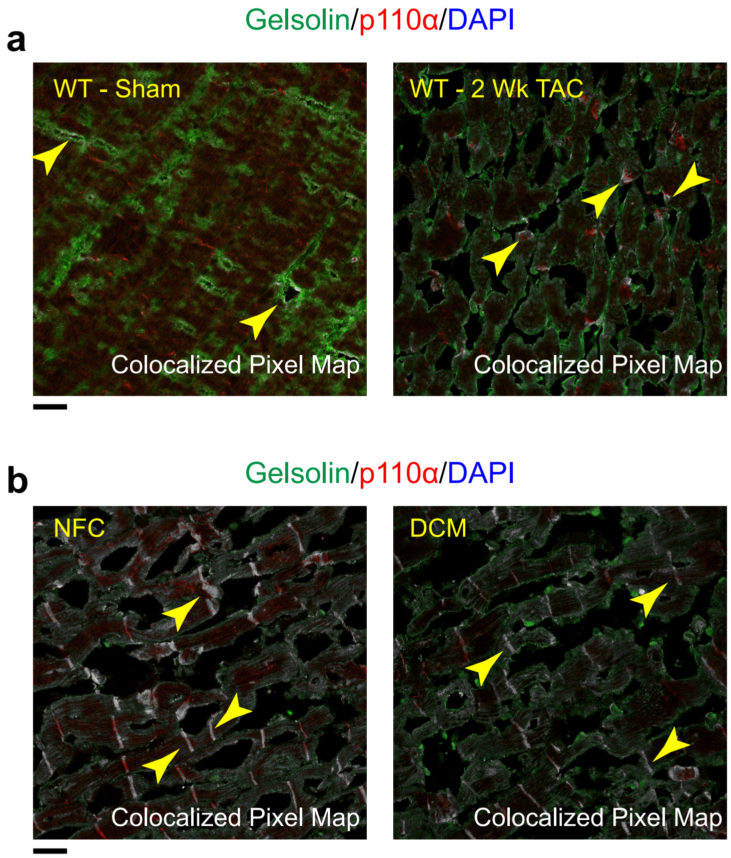

Supplementary Figure 7. p110α and gelsolin are spatially co-localized in human and murine hearts. **a**-**b** Co-localization pixel maps of representative immunofluorescence staining images showing spatial co-localization of p110α and gelsolin in murine (a) and human (b) hearts. Arrowheads indicate the co-localization areas (white pixels in images). Biological replicates: n=4. Scale bars show 25 μm (a & b).

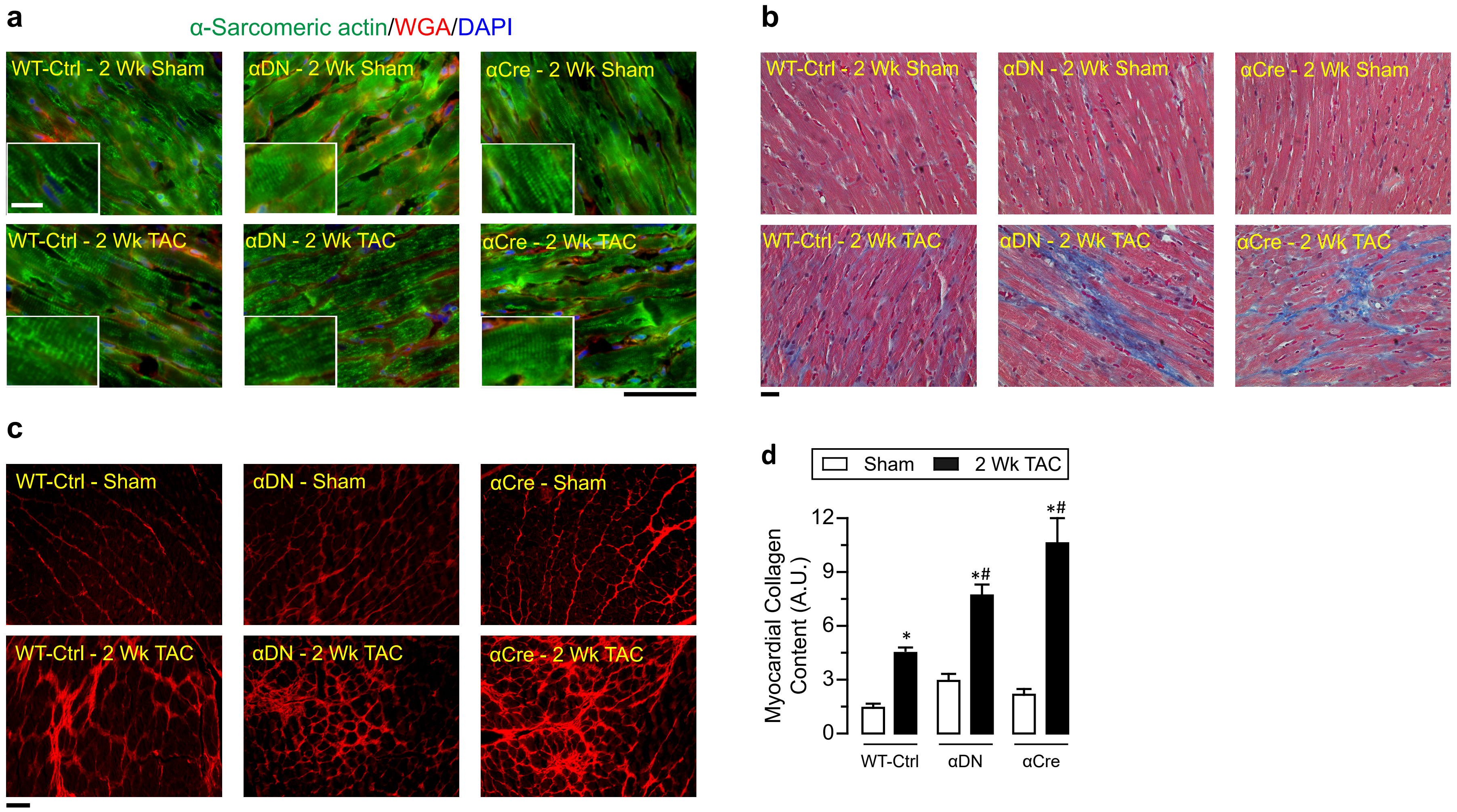

Supplementary Figure 8. Loss of p110α does not affect thin filament structure but leads to greater cardiac fibrosis in response to pressure overload. **a** Representative immunofluorescence images (a), with high magnification inserts, showing intact sarcomeric actin structure indicating preserved thin filament in all the groups. **b-d** Representative Masson trichrome staining (b) and picro-sirius red staining images (c) and quantification for myocardial collagen content (d) showing greater increase in cardiac fibrosis in the p110α transgenic mice compared with the WT control mice in response to pressure overload. Data represent means ± s.e.m. *P<0.05 compared with the respective control groups (Sham), #P<0.05 compared with WT-Ctrl – 2 Wk TAC group as determined by one-way ANOVA analysis (d). Biological replicates: n=4. Scale bars show 50 μm (a), 10 μm (high magnification inserts in a) and 25 μm (b, c).

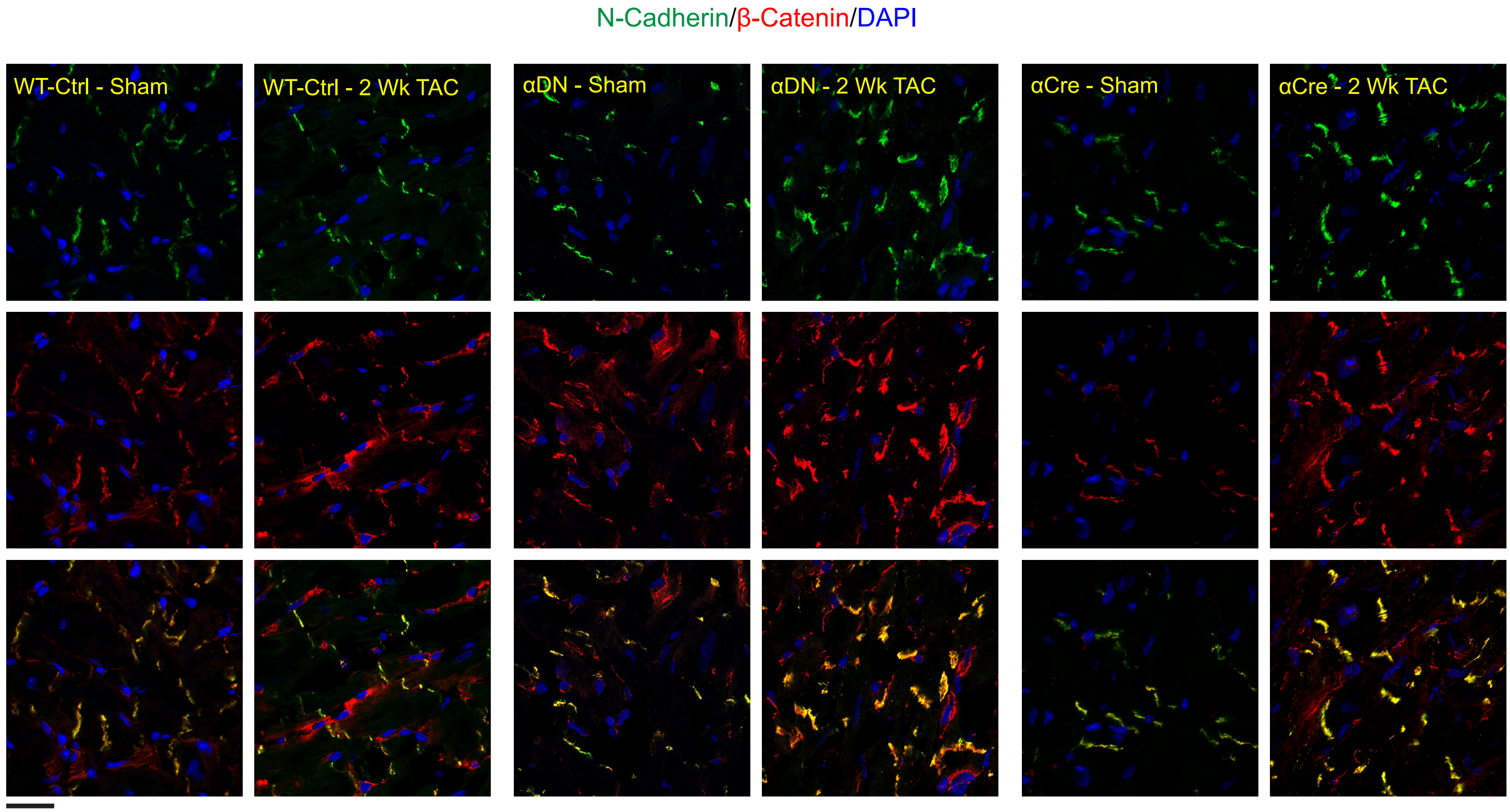

Supplementary Figure 9. Greater upregulation of mechano-sensors in p110α transgenic hearts in response to pressure overload-induced biomechanical stress. Representative IF images showing greater upregulation of myocardial levels of N-Cadherin and β-catenin, mechano-sensors at cell-cell junctions, in the p110α transgenic hearts in response to pressure overload induced biomechanical stress. Biological replicates: n=4. Scale bar shows 25 μm.

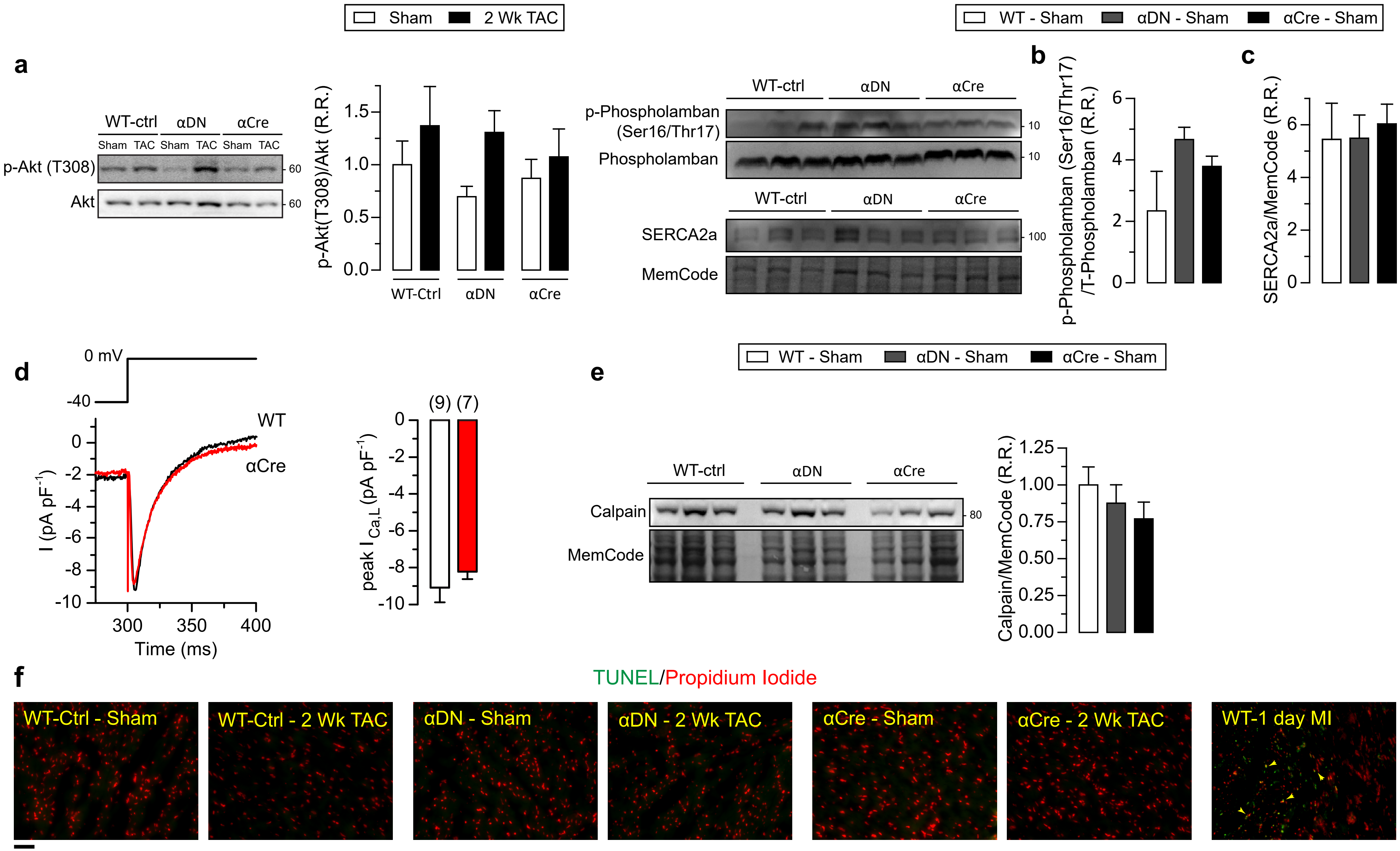

Supplementary Figure 10. Loss of p110α kinase activity does not alter the levels of calcium regulating proteins and calcium currents and does not predispose the myocardium to apoptosis in response to pressure overload. **a** Western blot analysis showing unchanged level of phosphorylation of Akt (T308 residue) in p110α transgenic and WT control hearts in response to pressure overload. **b-d** Western blot analyses showing unaltered levels of p-phospholamban (b), SERCA2a (c) resulting in unchanged L-type Ca^{2+} current (peak $I_{Ca,L}$) (d) in myocytes from p110α transgenic and WT hearts. **e** Western blot analyses showing unaltered levels of calpain (e) in p110α transgenic and WT hearts. **f** Representative TUNEL staining images showing no detectable apoptotic nuclei in p110α transgenic and WT control hearts. WT murine heart from 1 day-post LAD ligation-induced myocardial infarction was used as a positive control for TUNEL assay. Data represent means ± s.e.m. Biological replicates: n=4 (a-c, e-f) and n=6 (d). Scale bar shows 50 µm (f).

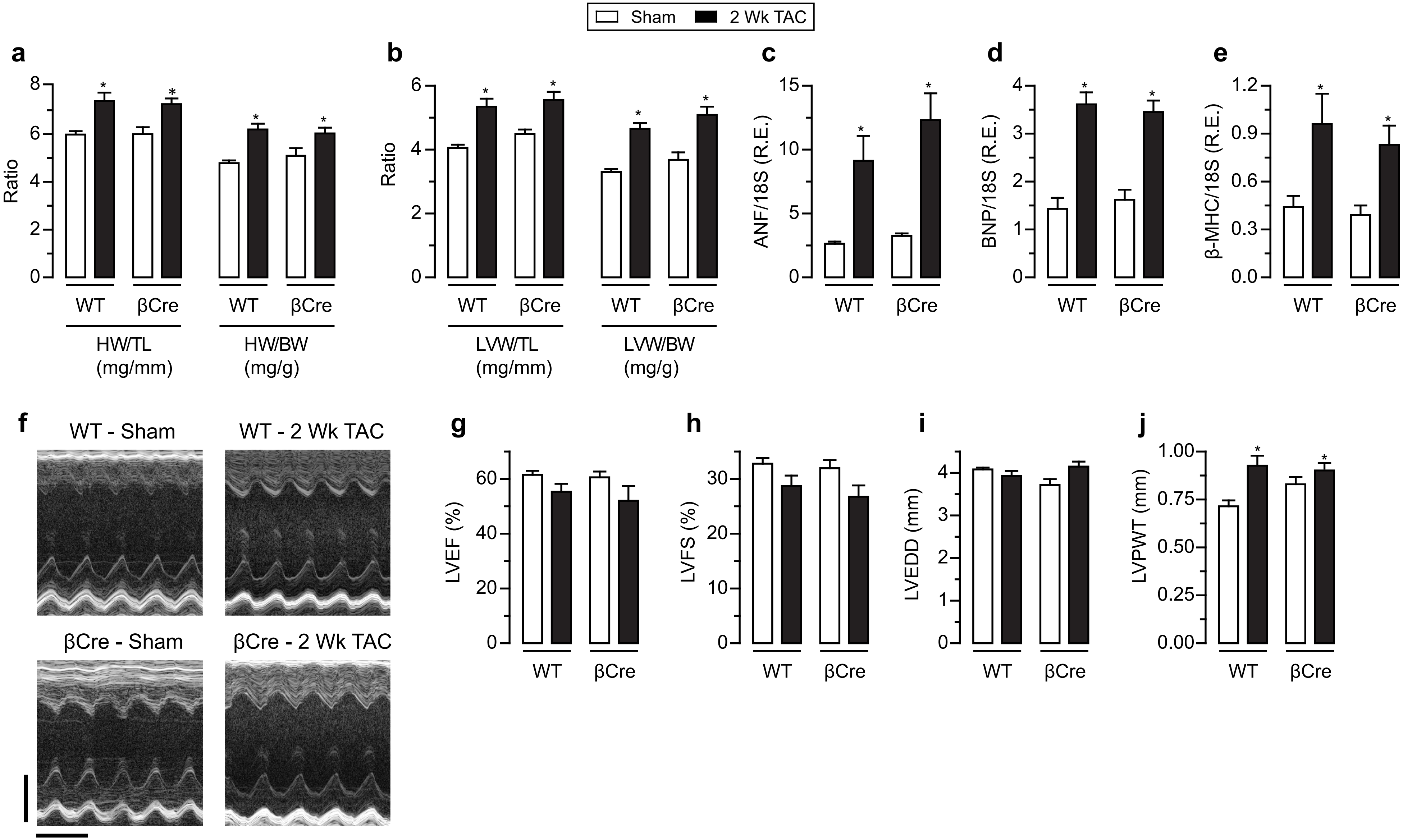

Supplementary Figure 11. Equivalent cardiac hypertrophy and cardiac dysfunction in p110β$^{flx/flx}$ Cre and WT mice in response to pressure-overload. **a-b** Heart weight and left ventricle (LV) ratios to tibial length and body weight showing equivalent cardiac hypertrophy in PI3Kα$^{flx/flx}$ No Cre (WT) and PI3Kα$^{flx/flx}$ Cre (βCre) hearts in response to pressure-overload. **c-e** mRNA expression analysis showing equivalent increase in the expression of cardiac disease markers including atrial natriuretic factor (ANF; c), brain natriuretic peptide (BNP; d) and β-myosin heavy chain (β-MHC; e) in WT and βCre hearts in response to pressure-overload. **f-j** Echocardiographic assessment of heart function using M-mode images of left ventricle (LV; f) and quantitative evaluation of cardiac function showing equivalent cardiac dysfunction and cardiac hypertrophy in WT and βCre hearts in response to pressure-overload as seen in the LV ejection fraction (LVEF; g), LV fractional shortening (LVFS; h), LV end-diastolic dimension (LVEDD; i), and LV posterior wall thickness (LVPWT; j). Data represent means ± s.e.m. *P<0.05 compared with the respective control groups (Sham) as determined by one-way ANOVA analysis (a-e, j). Biological replicates: n=12 (a-b, f-j) and n=10 (c-e). Scale bars show 2 mm (y-axis of f) and 200 ms (x-axis of f).

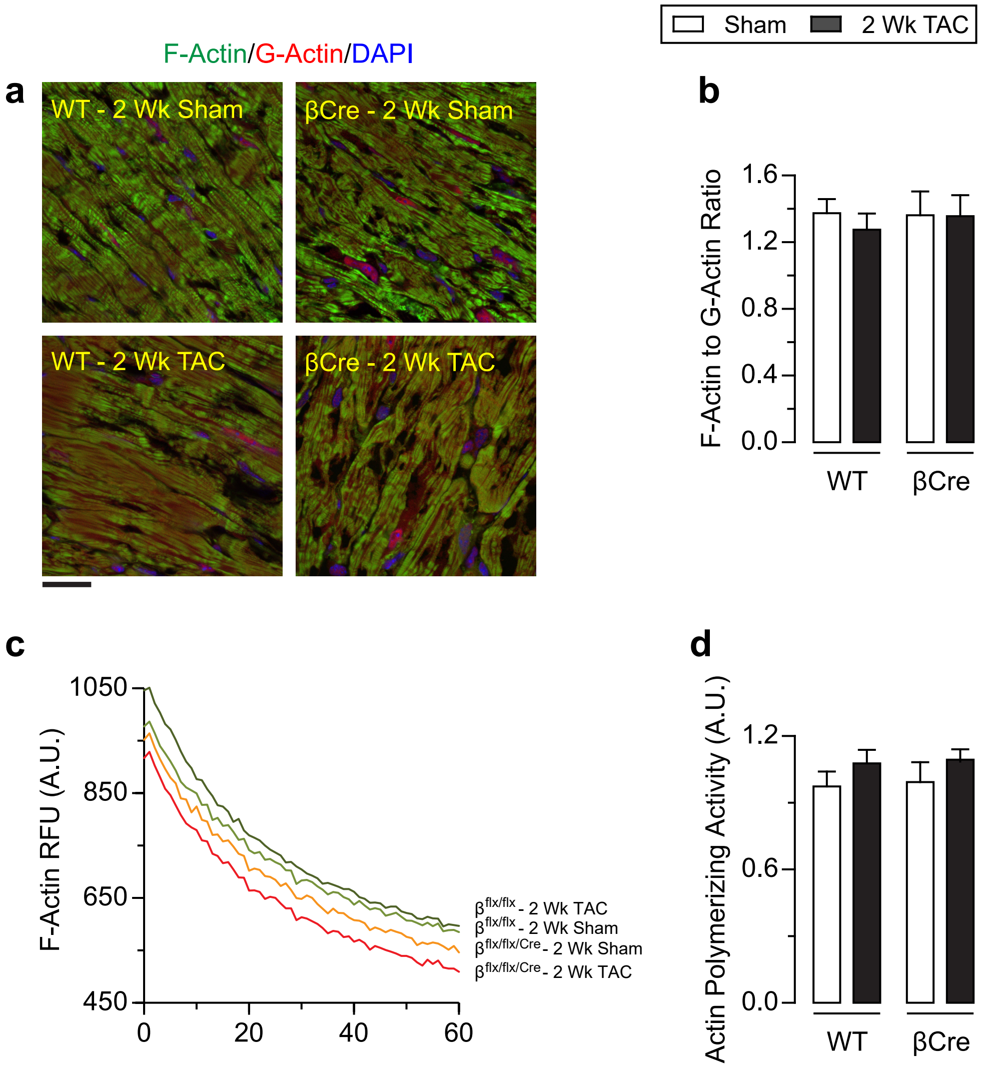

Supplementary Figure 12. Unchanged cytoskeletal remodeling in the PI3K$\beta^{flx/flx}$ Cre hearts in response to pressure-overload. **a-b** Representative phalloidin (F-actin) and DNAseI (G-actin) staining images and F-actin to G-actin ratio showing unaltered F/G-actin ratio in βCre and WT hearts in response to pressure-overload. **c-d** Fluorescence time courses of actin depolymerization assay (c) showing unchanged actin-severing activity (d) in the βCre and WT hearts in response to 2 weeks pressure-overload. Data represent means ± s.e.m. Biological replicates: n=4 (a-b) and n=6 (c-d). Scale bar shows 25 μm (a).

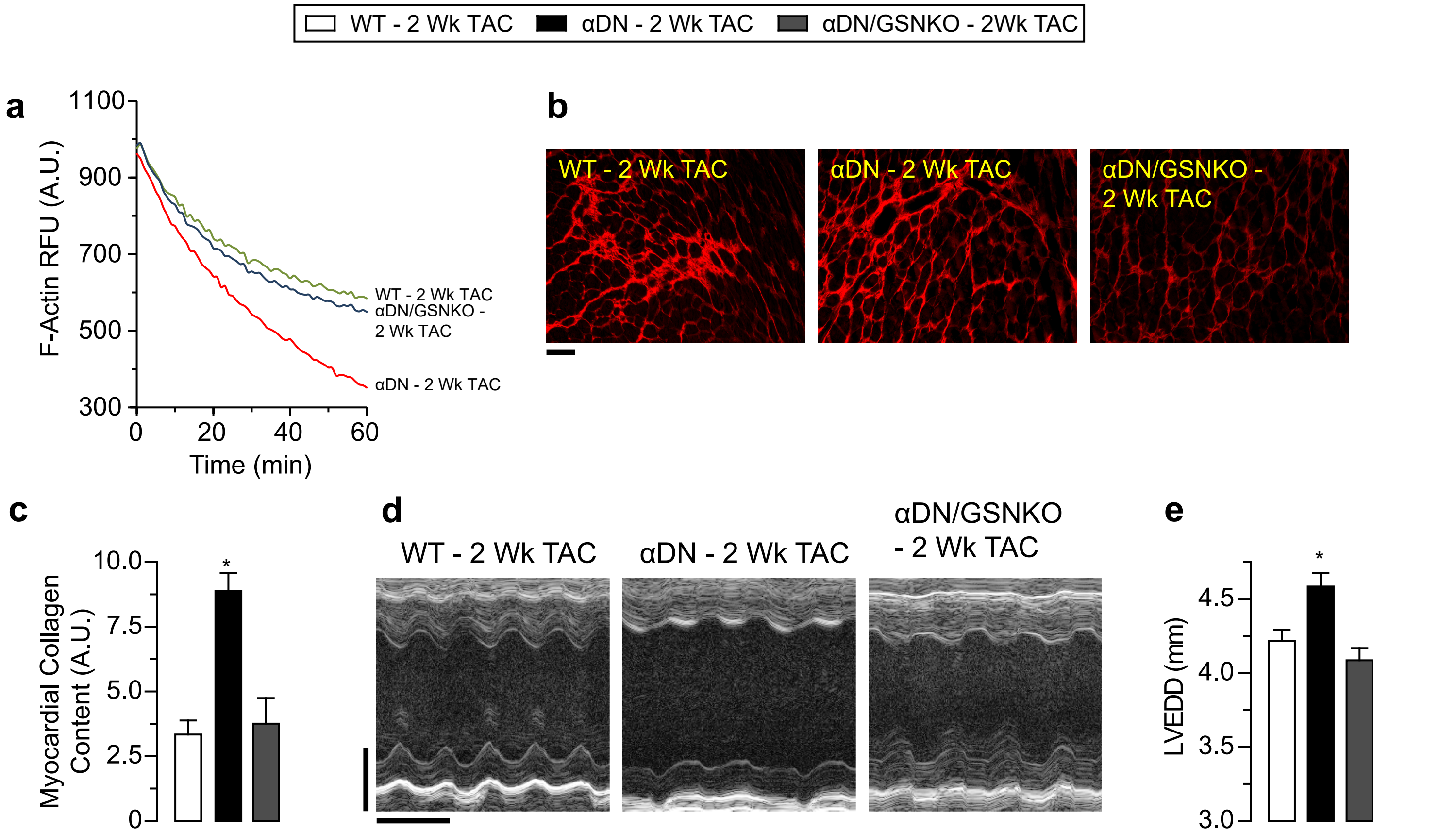

Supplementary Figure 13. Loss of gelsolin attenuates actin-severing activity, cardiac fibrosis, and cardiac dysfunction in response to pressure overload-induced biomechanical stress in p110α transgenic mice. **a** Actin depolymerization assay showing loss of gelsolin resulting in mitigation of actin severing activity in the PI3KαDN LVs in response to pressure-overload. **b-c** Picro-sirius red staining images (b) and quantification of myocardial collagen content (c) showing attenuation of cardiac fibrosis in response to pressure overload with loss of gelsolin. **d-e** Echocardiographic assessment of heart function using M-mode images of left ventricle (LV; d) and quantitative evaluation of cardiac dimensions showing ventricular dilation (e) in the αDN hearts in response to pressure overload, which was attenuated with the loss of gelsolin. Data represent means ± s.e.m. *P<0.05 compared to all the groups as determined by one-way ANOVA analysis (c, e). Biological replicates: n=6 (a), n=4 (b-c) and n=12 (d-e). Scale bars show 25 μm (b), 2 mm (y-axis of d) and 200 ms (x-axis of d).

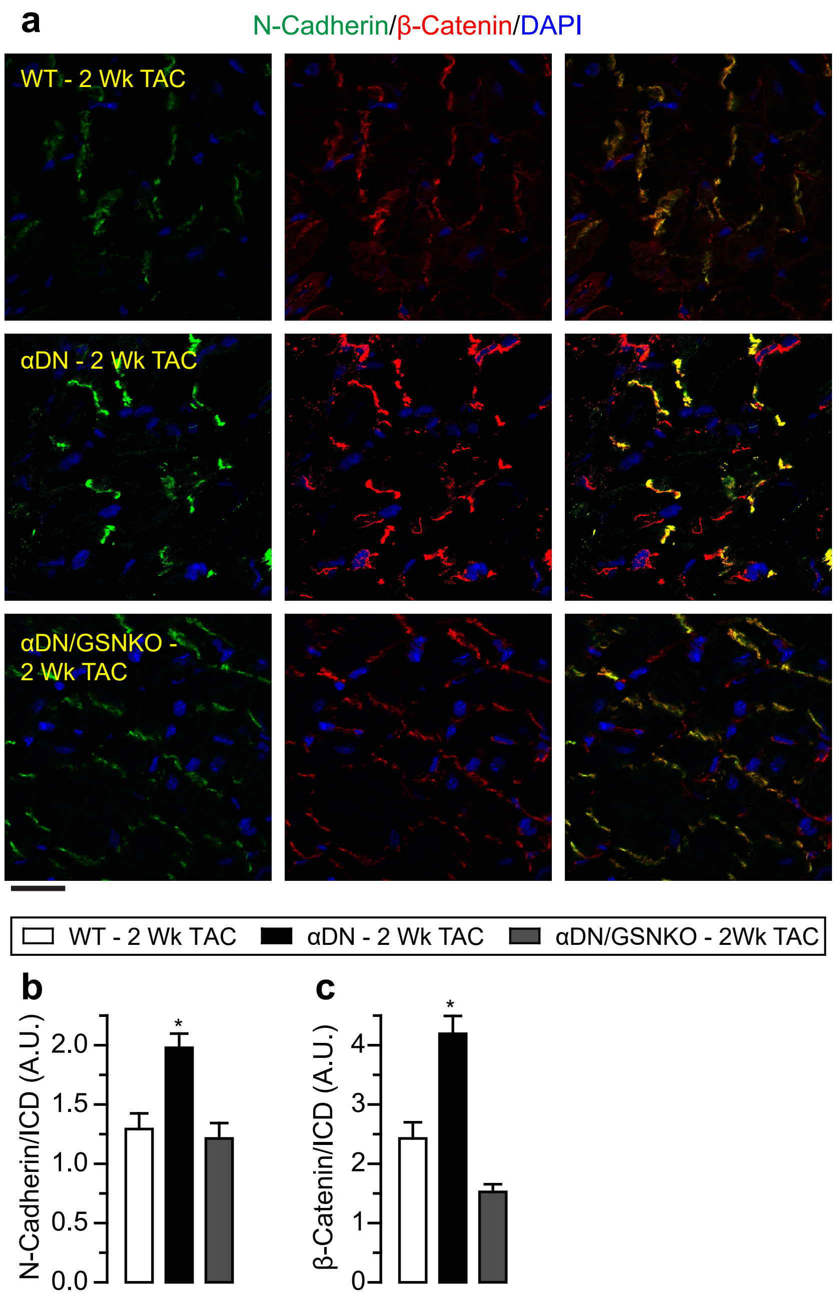

Supplementary Figure 14. Loss of gelsolin attenuates the biomechanical stress induced upregulation of N-cadherin and β-catenin in p110α transgenic mice. **a-c** Representative immunofluorescence (IF) staining images of N-cadherin and β-catenin showing marked increase in N-cadherin (b) and β-catenin (c) protein levels in PI3KαDN left ventricles (LVs) in response to pressure overload for 2 weeks, where loss of gelsolin preserved the pressure-overload induced upregulation of these adherens junction proteins. Data represent means ± s.e.m. *$P<0.05$ compared to all the groups as determined by one-way ANOVA analysis (b, c). Biological replicates: n=4. Scale bar shows 50 μm (a).

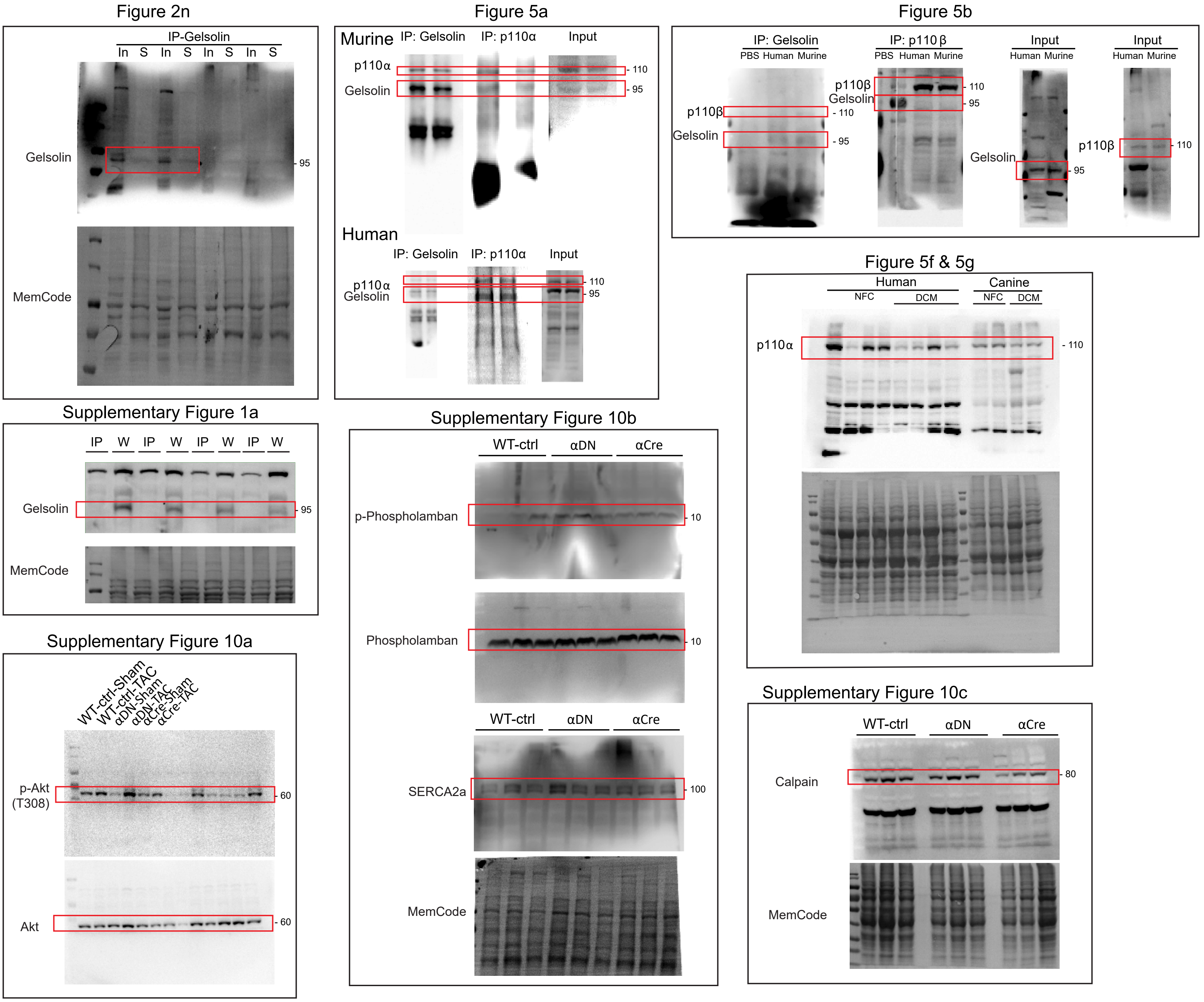

Supplementary Figure 15. Uncropped Western blots showing molecular weight markers from Figures 2n, 5a, and 5b and Supplementary Figures 1a, 10a, 10b, and 10c .

Supplementary References

1. Bodiga, S., *et al.* Enhanced susceptibility to biomechanical stress in ACE2 null mice is prevented by loss of the p47(phox) NADPH oxidase subunit. *Cardiovascular research* **91**, 151-161 (2011).
2. Guo, D., *et al.* Loss of PI3Kgamma enhances cAMP-dependent MMP remodeling of the myocardial N-cadherin adhesion complexes and extracellular matrix in response to early biomechanical stress. *Circ. Res.* **107**, 1275-1289 (2010).
3. Graupera, M., *et al.* Angiogenesis selectively requires the p110alpha isoform of PI3K to control endothelial cell migration. *Nature* **453**, 662-666 (2008).
4. Li, G.H., *et al.* Gelsolin regulates cardiac remodeling after myocardial infarction through DNase I-mediated apoptosis. *Circulation research* **104**, 896-904 (2009).
5. Patel, V.B., *et al.* Loss of p47phox subunit enhances susceptibility to biomechanical stress and heart failure because of dysregulation of cortactin and actin filaments. *Circ Res* **112**, 1542-1556 (2013).
6. Zhong, J.C., *et al.* Angiotensin-converting enzyme 2 suppresses pathological hypertrophy, myocardial fibrosis, and cardiac dysfunction. *Circulation* **122**, 717-728 (2010).
7. Patel, V.B., *et al.* Loss of angiotensin-converting enzyme-2 exacerbates diabetic cardiovascular complications and leads to systolic and vascular dysfunction: a critical role of the angiotensin II/AT1 receptor axis. *Circ Res* **110**, 1322-1335 (2012).
8. Basu, R., *et al.* Type 1 diabetic cardiomyopathy in the Akita (Ins2WT/C96Y) mouse model is characterized by lipotoxicity and diastolic dysfunction with preserved systolic function. *Am J Physiol Heart Circ Physiol* **297**, H2096-2108 (2009).
9. Patel, V.B., *et al.* ACE2 Deficiency Worsens Epicardial Adipose Tissue Inflammation and Cardiac Dysfunction in Response to Diet-Induced Obesity. *Diabetes* **65**, 85-95 (2016).
10. Wang, W., *et al.* Loss of Apelin exacerbates myocardial infarction adverse remodeling and ischemia-reperfusion injury: therapeutic potential of synthetic Apelin analogues. *J Am Heart Assoc* **2**, e000249 (2013).
11. Zhong, J., *et al.* Angiotensin-converting enzyme 2 suppresses pathological hypertrophy, myocardial fibrosis, and cardiac dysfunction. *Circulation* **122**, 717-728, 718 p following 728 (2010).
12. Nag, S., *et al.* Ca2+ binding by domain 2 plays a critical role in the activation and stabilization of gelsolin. *Proc Natl Acad Sci U S A* **106**, 13713-13718 (2009).
13. Narayan, K., *et al.* Activation in isolation: exposure of the actin-binding site in the C-terminal half of gelsolin does not require actin. *FEBS Lett* **552**, 82-85 (2003).
14. Choe, H., *et al.* The calcium activation of gelsolin: insights from the 3A structure of the G4-G6/actin complex. *Journal of molecular biology* **324**, 691-702 (2002).
15. Sali, A. & Blundell, T.L. Comparative protein modelling by satisfaction of spatial restraints. *Journal of molecular biology* **234**, 779-815 (1993).
16. Sali, A., Potterton, L., Yuan, F., van Vlijmen, H. & Karplus, M. Evaluation of comparative protein modeling by MODELLER. *Proteins* **23**, 318-326 (1995).
17. Mills, N. ChemDraw Ultra 10.0 CambridgeSoft, 100 CambridgePark Drive, Cambridge, MA 02140. www.cambridgesoft.com. Commercial Price: $1910 for download, $2150 for CD-ROM; Academic Price: $710 for download, $800 for CD-ROM. *Journal of the American Chemical Society* **128**, 13649-13650 (2006).
18. Trott, O. & Olson, A.J. AutoDock Vina: improving the speed and accuracy of docking with a new scoring function, efficient optimization, and multithreading. *Journal of computational chemistry* **31**, 455-461 (2010).
19. Urosev, D., Ma, Q., Tan, A.L., Robinson, R.C. & Burtnick, L.D. The structure of gelsolin bound to ATP. *Journal of molecular biology* **357**, 765-772 (2006).

20. Emsley, P. & Cowtan, K. Coot: model-building tools for molecular graphics. *Acta crystallographica. Section D, Biological crystallography* **60**, 2126-2132 (2004).
21. Van Der Spoel, D.*, et al.* GROMACS: fast, flexible, and free. *Journal of computational chemistry* **26**, 1701-1718 (2005).
22. Schuttelkopf, A.W. & van Aalten, D.M. PRODRG: a tool for high-throughput crystallography of protein-ligand complexes. *Acta crystallographica. Section D, Biological crystallography* **60**, 1355-1363 (2004).
23. Miyamoto, S. & Kollman, P.A. Settle: An analytical version of the SHAKE and RATTLE algorithm for rigid water models. *Journal of computational chemistry* **13**, 952-962 (1992).
24. Hess, B., Bekker, H., Berendsen, H.J.C. & Fraaije, J.G.E.M. LINCS: A linear constraint solver for molecular simulations. *Journal of computational chemistry* **18**, 1463-1472 (1997).
25. Darden, T., York, D. & Pedersen, L. Particle mesh Ewald: An N·log(N) method for Ewald sums in large systems. *The Journal of Chemical Physics* **98**, 10089-10092 (1993).
26. Ryckaert, J.-P., Ciccotti, G. & Berendsen, H.J.C. Numerical integration of the cartesian equations of motion of a system with constraints: molecular dynamics of n-alkanes. *Journal of Computational Physics* **23**, 327-341 (1977).
27. Lill, M.A. & Danielson, M.L. Computer-aided drug design platform using PyMOL. *J Comput Aided Mol Des* **25**, 13-19 (2011).
28. Humphrey, W., Dalke, A. & Schulten, K. VMD: visual molecular dynamics. *Journal of molecular graphics* **14**, 33-38, 27-38 (1996).
29. Pettersen, E.F.*, et al.* UCSF Chimera--a visualization system for exploratory research and analysis. *Journal of computational chemistry* **25**, 1605-1612 (2004).
30. Vaught, A. Graphing With Gnuplot And Xmgr (Http://Dl.Acm.Org/Citation.Cfm?Id=326334); Linux Journal. (1996).
31. Franklin, J., Koehl, P., Doniach, S. & Delarue, M. MinActionPath: maximum likelihood trajectory for large-scale structural transitions in a coarse-grained locally harmonic energy landscape. *Nucleic Acids Res* **35**, W477-482 (2007).
32. Lipfert, J., Franklin, J., Wu, F. & Doniach, S. Protein misfolding and amyloid formation for the peptide GNNQQNY from yeast prion protein Sup35: simulation by reaction path annealing. *Journal of molecular biology* **349**, 648-658 (2005).
33. Eastman, P., Grønbech-Jensen, N. & Doniach, S. Simulation of protein folding by reaction path annealing. *The Journal of Chemical Physics* **114**, 3823-3841 (2001).
34. Amadei, A., Ceruso, M.A. & Di Nola, A. On the convergence of the conformational coordinates basis set obtained by the essential dynamics analysis of proteins' molecular dynamics simulations. *Proteins* **36**, 419-424 (1999).
35. Amadei, A., Linssen, A.B. & Berendsen, H.J. Essential dynamics of proteins. *Proteins* **17**, 412-425 (1993).
36. Paul, M., Hazra, M., Barman, A. & Hazra, S. Comparative molecular dynamics simulation studies for determining factors contributing to the thermostability of chemotaxis protein "CheY". *J Biomol Struct Dyn* **32**, 928-949 (2014).
37. Patel, V.B.*, et al.* Cardioprotective effects mediated by angiotensin II type 1 receptor blockade and enhancing angiotensin 1-7 in experimental heart failure in angiotensin-converting enzyme 2-null mice. *Hypertension (Dallas, Tex. : 1979)* **59**, 1195-1203 (2012).
